# Supplementary material for: Directing microbial co-culture composition using cybernetic control
Source: Cell Rep Methods. 2025 Mar 24;5(3):101009. doi: 10.1016/j.crmeth.2025.101009 (PMC12049730; doi:10.1016/j.crmeth.2025.101009)
Supplement: Document S1. Figures S1–S7 [file mmc1.pdf]

Cell Reports Methods, Volume *5*

Supplemental information

Directing microbial co-culture composition using cybernetic control

Ting An Lee, Jan Morlock, John Allan, and Harrison Steel

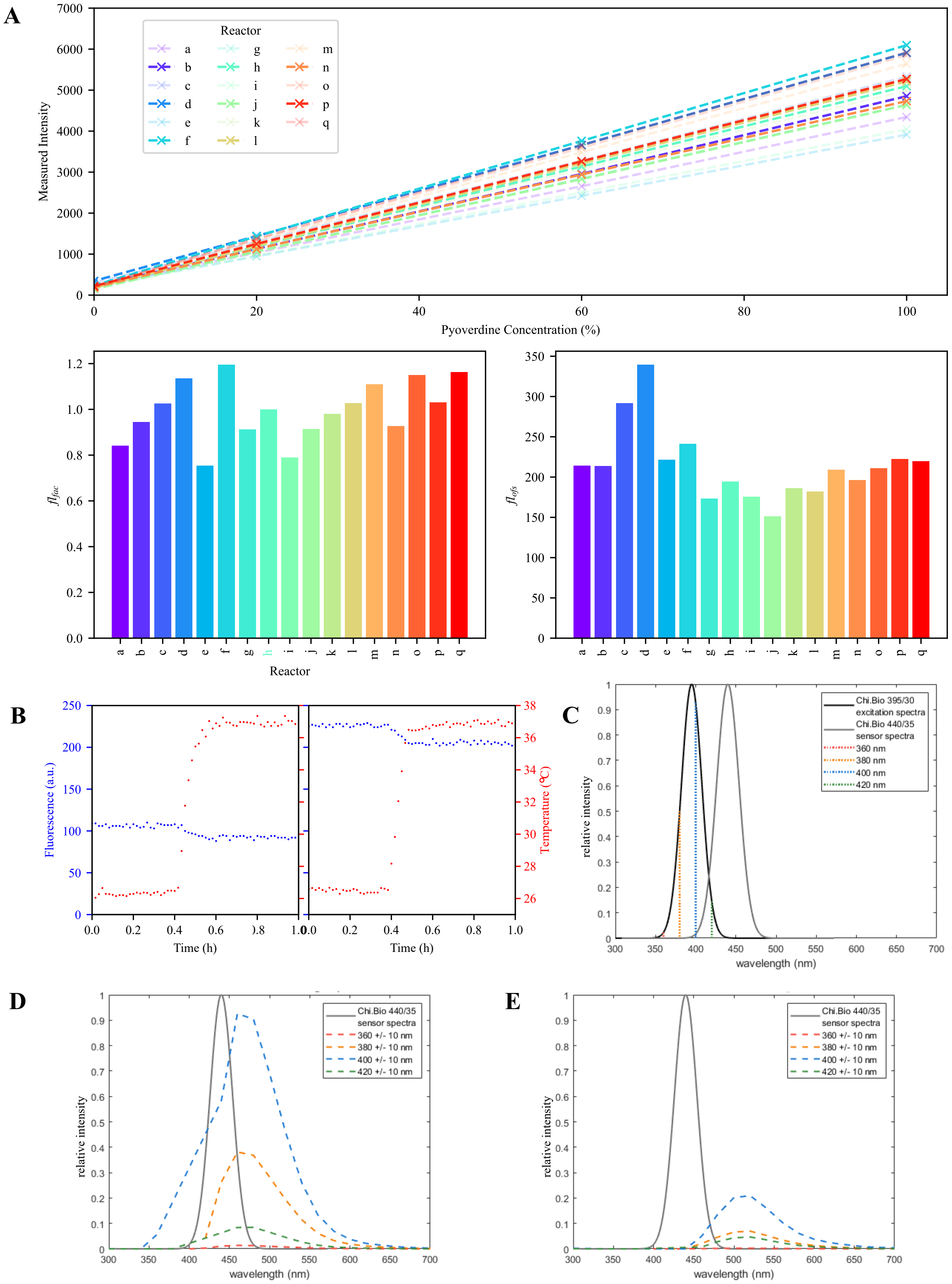
A
Reactor
a b c d e f g h i j k l m n o p q
Measured Intensity
Pyoverdine Concentration (%)
fl_{fac}
fl_{ofs}
Reactor
B
Fluorescence (a.u.)
Temperature (°C)
Time (h)
C
Chi.Bio 395/30 excitation spectra
Chi.Bio 440/35 sensor spectra
360 nm
380 nm
400 nm
420 nm
relative intensity
wavelength (nm)
D
Chi.Bio 440/35 sensor spectra
360 +/- 10 nm
380 +/- 10 nm
400 +/- 10 nm
420 +/- 10 nm
relative intensity
wavelength (nm)
E
Chi.Bio 440/35 sensor spectra
360 +/- 10 nm
380 +/- 10 nm
400 +/- 10 nm
420 +/- 10 nm
relative intensity
wavelength (nm)

Figure S1: Bioreactor measurements for composition information, related to Figure 2. (A) Calibrating fluorescence readings in different bioreactors. Each reactor was given a unique identifier and then calibrated (top) by measuring fluorescence (a.u.) of a serial dilution of a filter-sterilised *Pseudomonas putida* (*P. putida*) monoculture (removing cells but maintaining fluorescent extracellular pyoverdine). Used excitation/excitation wavelengths of 395/$440\,\mathrm{nm}$ and diluted with M9 media. These measurements were used to calculate a factor fl_{fac} (bottom left) and offset fl_{ofs} (bottom right) for each reactor, where the factor is relative to reactor h. **(B) Effect of temperature on media and pyoverdine.** Reactor runs of just fresh media (M9 + casamino acid (CAA), left) and a filtered *P. putida* monoculture (i.e. M9 + CAA + pyoverdine, right). The negligible ($<$30 a.u.) change in fluorescence across a temperature range larger than conditions used in experiments for both plain media and media with pyoverdine indicates that large changes in fluorescence observed in experiments with cells are due to changes in pyoverdine production. **(C) Fluorescence overlap accounting for hardware - measuring hardware spectra** Fluorescence overlap remains minimal even when taking into account the spectra of the Chi.Bio's 395 nm excitation LED, which has a full width half maximum (FWHM) of 30 nm, and the spectra of the 440 nm sensor, which has a FWHM of 35 nm. (Black curve) relative intensity of different wavelengths of light emitted by the Chi.Bio when the 395/30 nm laser is turned on, and (grey curve) relative intensity of wavelengths measured by the Chi.Bio's 440/35 nm sensor. **(D) Fluorescence overlap accounting for hardware - *P. putida*** Emission spectra of *P. putida* excited with different wavelengths of light (360, 380, 400, 420 nm) measured in a plate reader with a step size and bandwidth of 20 nm, scaled according to the intensity of different excitation wavelengths that result from turning on the 395/30 nm laser in A. The areas under the intersection of the Chi.Bio440/35 sensor spectra (grey curve) with the emission spectra thus represent the fluorescence of *P. putida* measured when turning on the 395/30 nm laser. **(E) Fluorescence overlap accounting for hardware - *Escherichia coli* (*E. coli*)** Same as D, but for *E. coli* (scaled emission spectra when excited at wavelengths of 360, 380, 400, 420 nm).

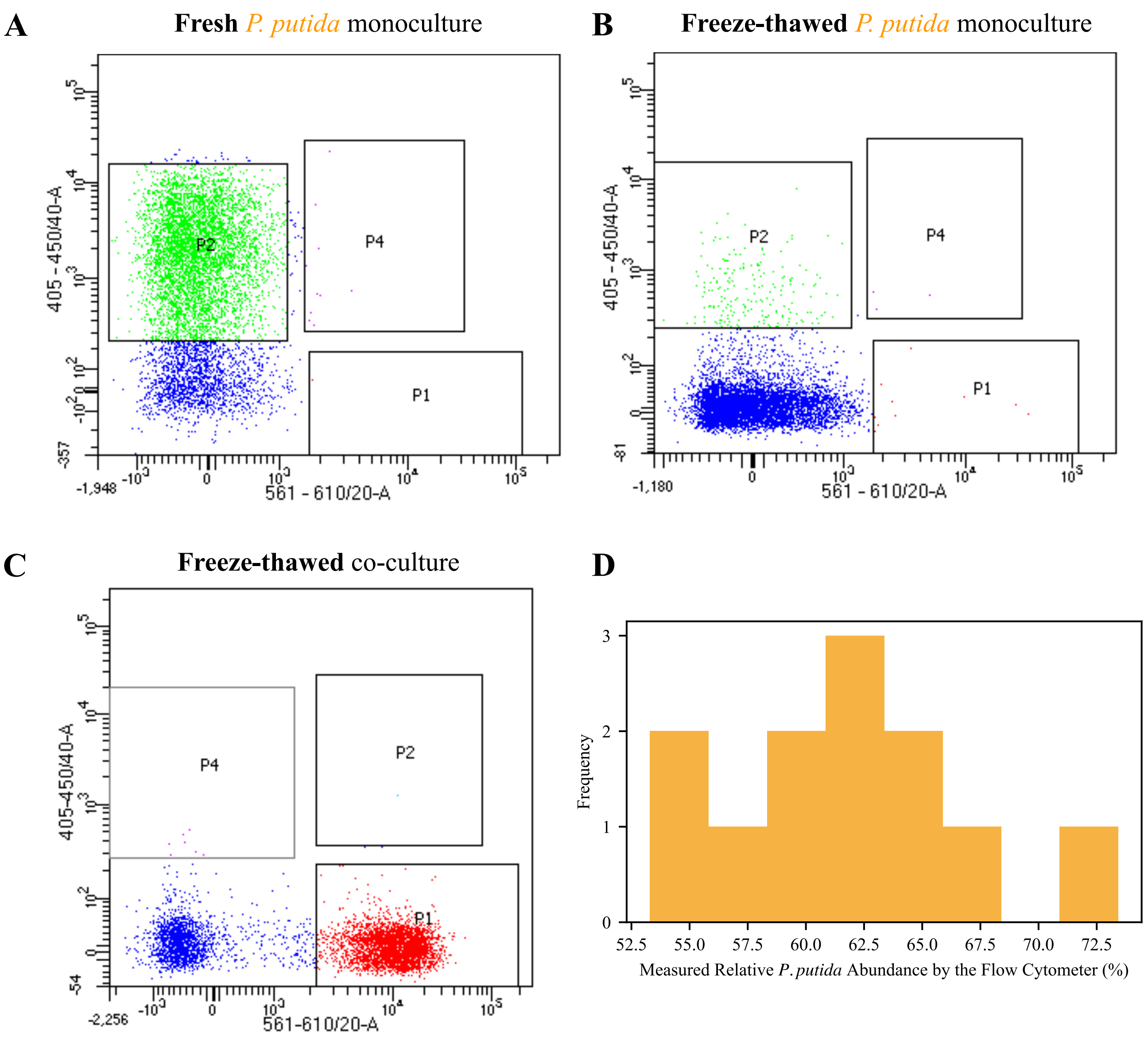

Figure S2: flow cytometry data, related to STAR Methods. Flow cytometry data. Freeze-thawing stored samples causes pyoverdine to leak out of cells. Flow cytometry gating of **fresh (A)/freeze-thawed (B) samples** from a *P. putida* monoculture, and a **freeze-thawed sample from a co-culture (C)**. Cells were first gated by forward and side scatter (not shown), and then by fluorescence intensity for pyoverdine (405 - 450/40-A) or red fluorescent protein (RFP) (561 - 610/20-A) wavelengths. Fresh *P. putida* has significant amounts of pyoverdine fluorescence, but almost is lost after freeze-thawing. In the flow cytometer, species are instead distinguished using the RFP's fluorescence, which persists after a freeze-thaw. **(D) Flow cytometer noise.** Histogram of twelve flow cytometer measurements of the same composition. This resulted in an estimated flow cytometry measurement uncertainty with a standard deviation of $5.3\,\%$.

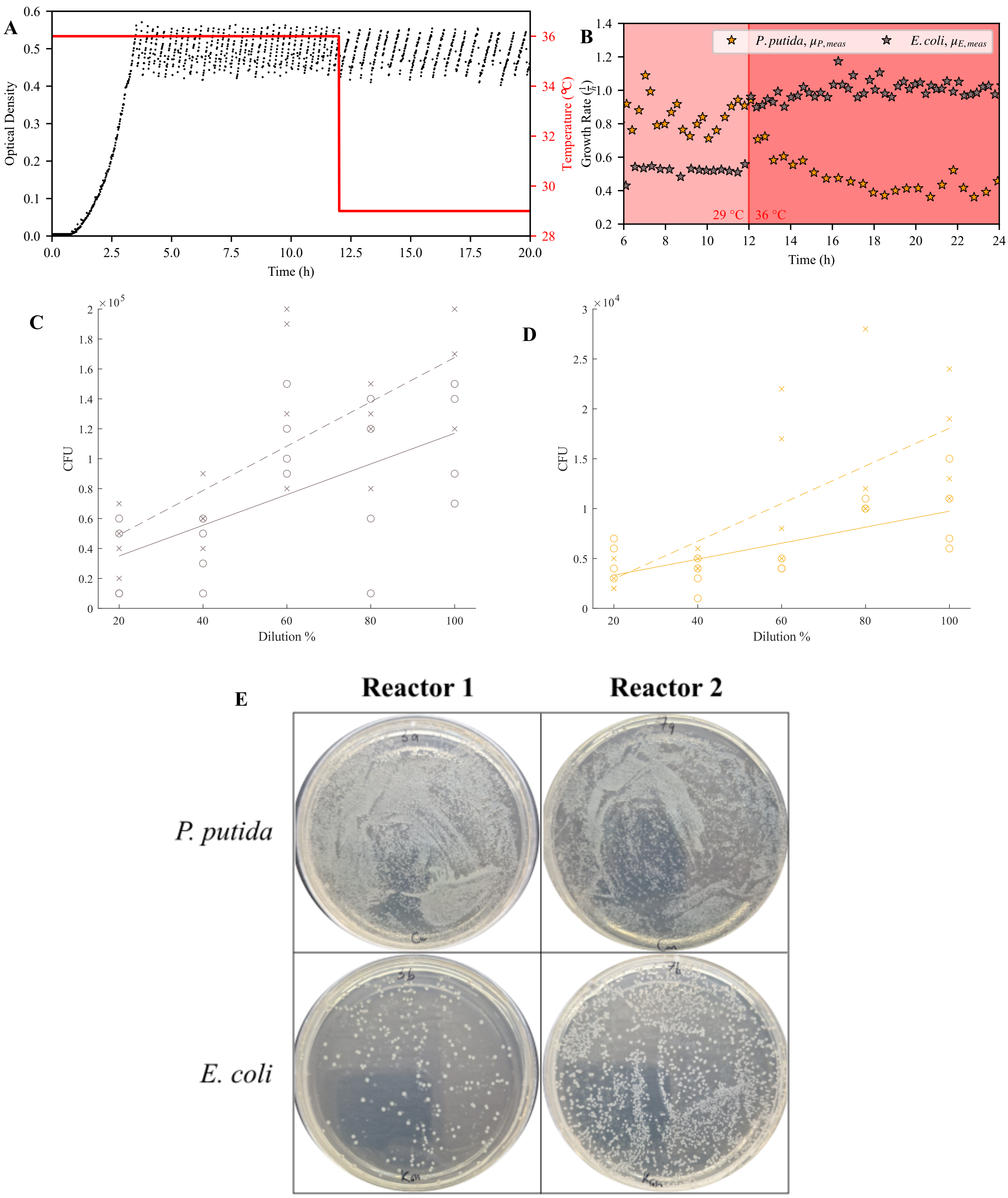
A
Optical Density
Temperature (°C)
Time (h)
B
P. putida, $\mu_{P,meas}$
E. coli, $\mu_{E,meas}$
Growth Rate ($\frac{1}{h}$)
29 °C
36 °C
Time (h)
C
$\times 10^5$
CFU
Dilution %
D
$\times 10^4$
CFU
Dilution %
E
Reactor 1
Reactor 2
P. putida
E. coli

Figure S3: growth dynamics and composition measurements, related to Figure 2. (A) OD dithering around setpoint. The total optical density (OD) (measured at 600_{nm}) of the culture dithers around a setpoint of 0.5, growing from 0 until it reaches 0.545, at which point fresh media is pumped in until the OD is diluted down to 0.455. This creates phases of dilution and growth from which we are able to calculate the growth rate of the mono or co-culture). When the temperature changes, the steepness of the curve during the growth period visibly changes as well. **(B) Change of the growth rates under a temperature change from $29\,°\mathrm{C}$ to $36\,°\mathrm{C}$.** Growth rates of *P. putida* and *E. coli* calculated from monocultures OD data, where dark red background = $36\,°\mathrm{C}$, light red background = $29\,°\mathrm{C}$. *P. putida* has a laggy response to temperature change in both directions. **Number of Colony Foming Units (CFUs) from a dilution series of *E. coli* (C) and *P. putida* (D) from bioreactor monoculture**. Diluted with $20\,\%$ intervals using PBS. Technical replicates at each dilution and biological replicates in different reactors vary, possibly due to noise in OD measurement. Flow cytometry, which measures ratios, is unaffected by this type of noise. **(E) Composition of biofilms.** Biofilms from a *P. putida* KT2440 (Cm resistance) and *E. coli* (Km resistance) co-cultures mixed at a 1:1 ratio and maintained at the 33.2C critical temperature. The biofilm in the reactors after overnight growth were scraped, resuspended in 100 ul PBS, and then spread onto plates with chloramphenicol or kanamycin. In both reactors, the chloramphenicol plates have semi-confluent growth, while the kanamycin plates have fewer colonies, indicating that the biofilm contains both species but consists primarily of *P. putida* colony forming units. Quantitative comparison *between* reactors is avoided, as the biofilm thickness and amount scraped was similar but not standardised. Controls of *P. putida* on kanamycin plates or *E. coli* on chloramphenicol plates have no colonies (not shown). Co-cultures with *P. putida* -lapA grown at the same time did not have visible biofilm, in line with monoculture behaviour.

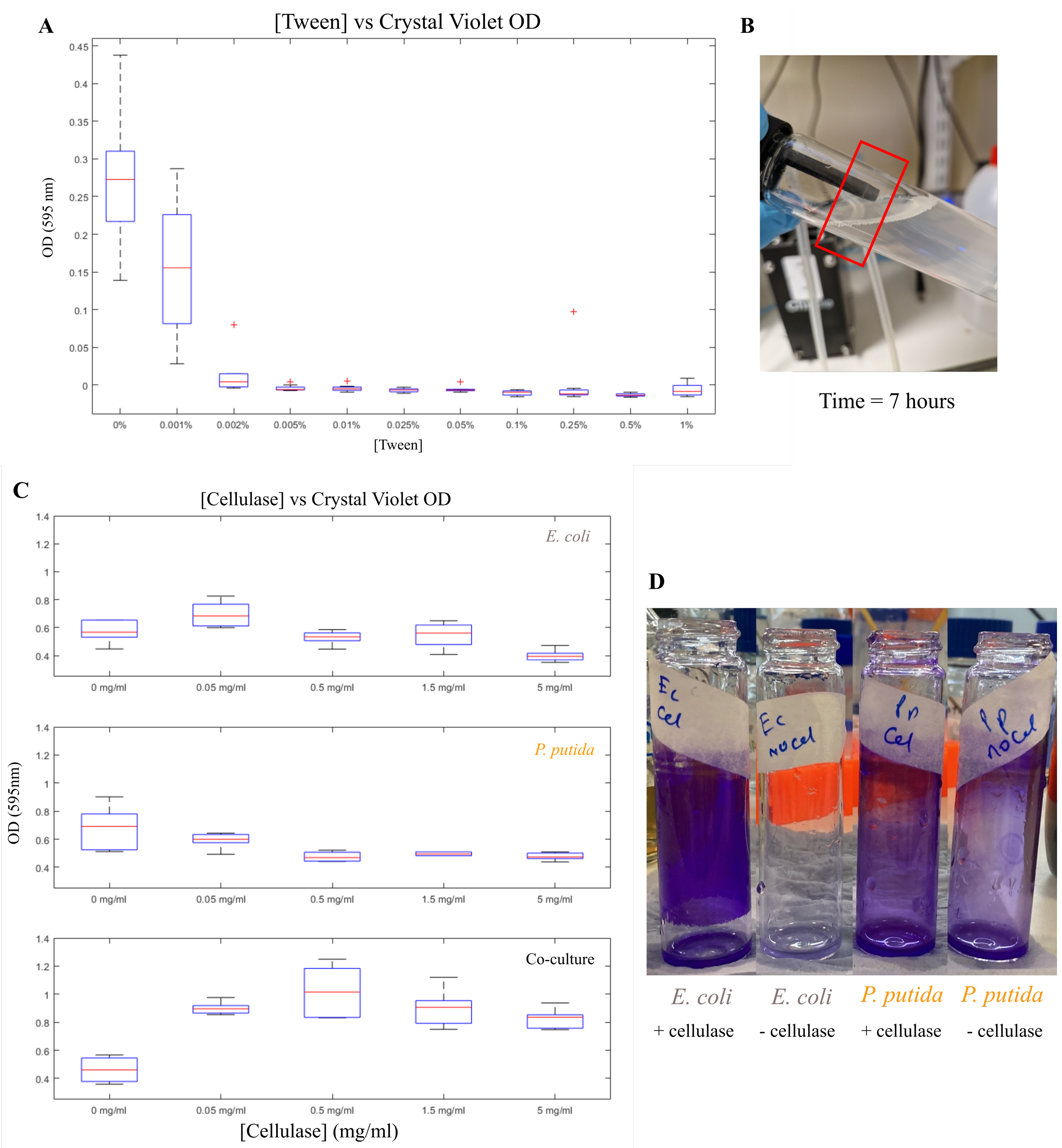
A
[Tween] vs Crystal Violet OD
OD (595 nm)
0.45
0.4
0.35
0.3
0.25
0.2
0.15
0.1
0.05
0
0%
0.001%
0.002%
0.005%
0.01%
0.025%
0.05%
0.1%
0.25%
0.5%
1%
[Tween]
B
Time = 7 hours
C
[Cellulase] vs Crystal Violet OD
OD (595nm)
1.4
1.2
1
0.8
0.6
0.4
E. coli
P. putida
Co-culture
0 mg/ml
0.05 mg/ml
0.5 mg/ml
1.5 mg/ml
5 mg/ml
[Cellulase] (mg/ml)
D
E. coli
+ cellulase
E. coli
- cellulase
P. putida
+ cellulase
P. putida
- cellulase

Figure S4: anti-biofilm measures, related to Figure 3 Polysorbate (Tween) 20 to prevent biofilms in 96 well plates. Range of concentrations of Tween 20 vs OD_{595} of ethanol-solubilised crystal violet used to stain the wells of 96 well plate growing *P. putida*. A higher OD = more biofilm. Compared to the no-Tween control, concentrations $>0.001\,\%$ have significantly less biofilm formation in plates. **(B) Polysorbate (Tween) 20 to prevent biofilms in bioreactor.** Monoculture of *P. putida* in a reactor vial after 7 hours of growth with $0.01\,\%$ Tween 20. A thin biofilm has still formed after 7 hours at the air-water interface, as highlighted in the red box. **(C) Cellulase to prevent biofilms in 96 well plates.** Range of concentrations of cellulase vs OD_{595} of ethanol-solubilised crystal violet used to stain the wells of 96 well plates growing *E. coli* (top), *P. putida* (middle), or both (bottom). A higher OD = more biofilm. Concentrations above $0.5\,\mathrm{mg\,mL^{-1}}$ had less *P. putida* biofilm in plates, while cellulase did not have a clear effect on *E. coli*. Co-cultures had more biofilm when cellulase was added, but not in a concentration-dependent way. **(D) Cellulase to prevent biofilms in bioreactor.** Monocultures grown in a reactor vial overnight with/without $0.5\,\mathrm{mg\,mL^{-1}}$ cellulase, where depth of purple indicates amount of biofilm. Cellulase caused *E. coli* to form an extremely thick biofilm, while it appeared to slightly inhibit *P. putida* biofilm formation.

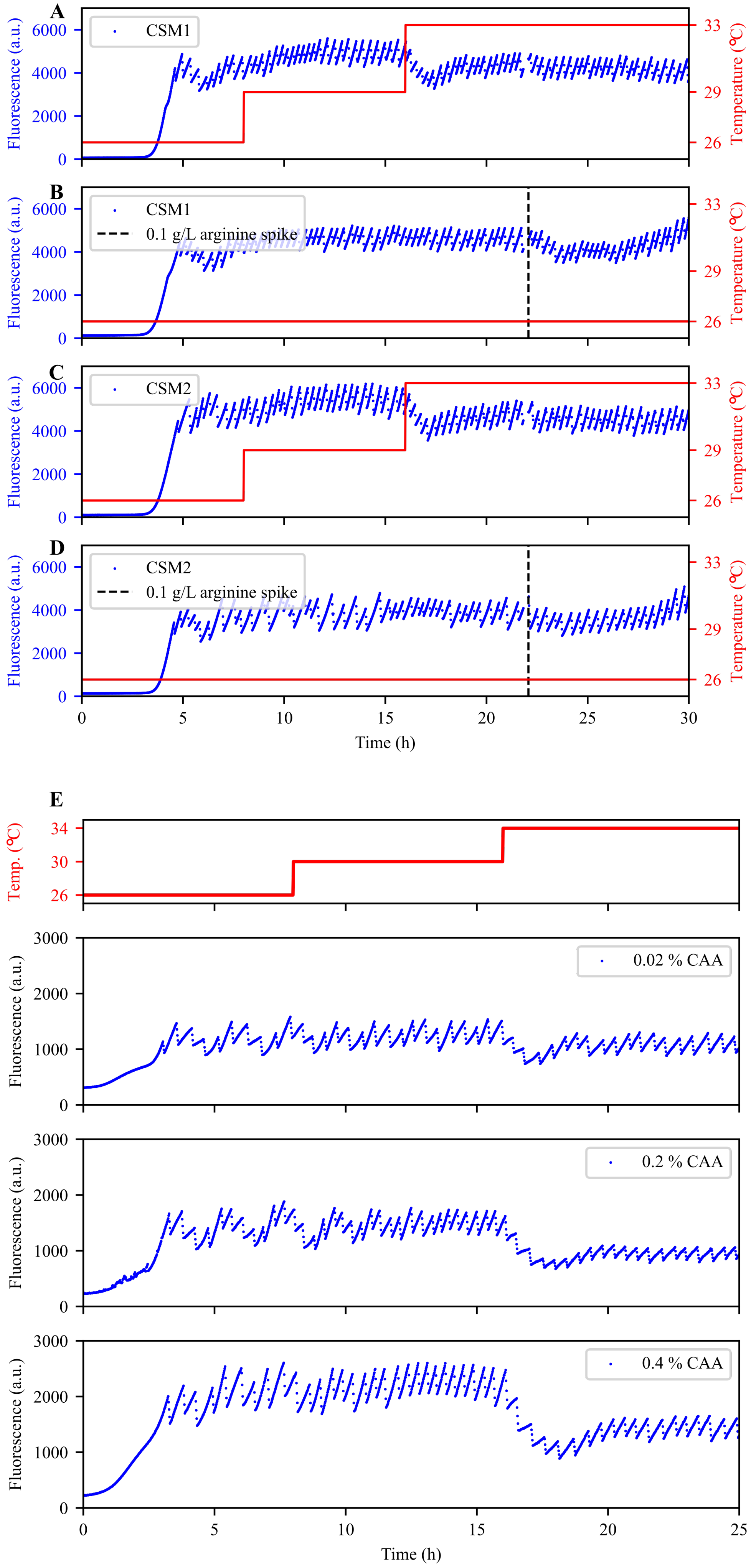

A
CSM1
B
CSM1
0.1 g/L arginine spike
C
CSM2
D
CSM2
0.1 g/L arginine spike
Fluorescence (a.u.)
Temperature (℃)
Time (h)
E
Temp. (℃)
0.02 % CAA
0.2 % CAA
0.4 % CAA
Fluorescence (a.u.)
Time (h)

Figure S5: effect of casamino acids (CAA) and complete synthetic amino acid mixtures (CSM) on oscillations, related to Figure 3. (A - D) Effect of different CSMs and arginine on oscillating fluorescence *P. putida* monocultures grown with double dropout Complete Synthetic Mixtures (- arginine and -tryptophan, both thought to influence pyoverdine production, from Formedium (see Key Resources Table). CSM1 (A, B) is supplemented with $100\,\mathrm{mg\,L^{-1}}$ serine, while CSM2 (C, D) is supplemented with $100\,\mathrm{mg\,L^{-1}}$ serine and glutamine. Runs B and D were spiked with $100\,\mathrm{mg\,L^{-1}}$ arginine at around time = 22 hours, which caused production to briefly drop before rising. **Effect of temperature on oscillatory behaviour of pyoverdine production.** Fluorescence is affected by the dithering behaviour of the culture (spiky increases/drops as the culture grows/is diluted)- this is not seen in the Fig. 3D/E, which were grown without dithering (i.e. very small dilutions whenever OD exceeded the setpoint, leading to an almost constant OD). Aside from the spikes, oscillations are also visible at $26\,^{\circ}\mathrm{C}$. The amplitude of the oscillations appears to be related to temperature, decreasing at warmer temperatures.

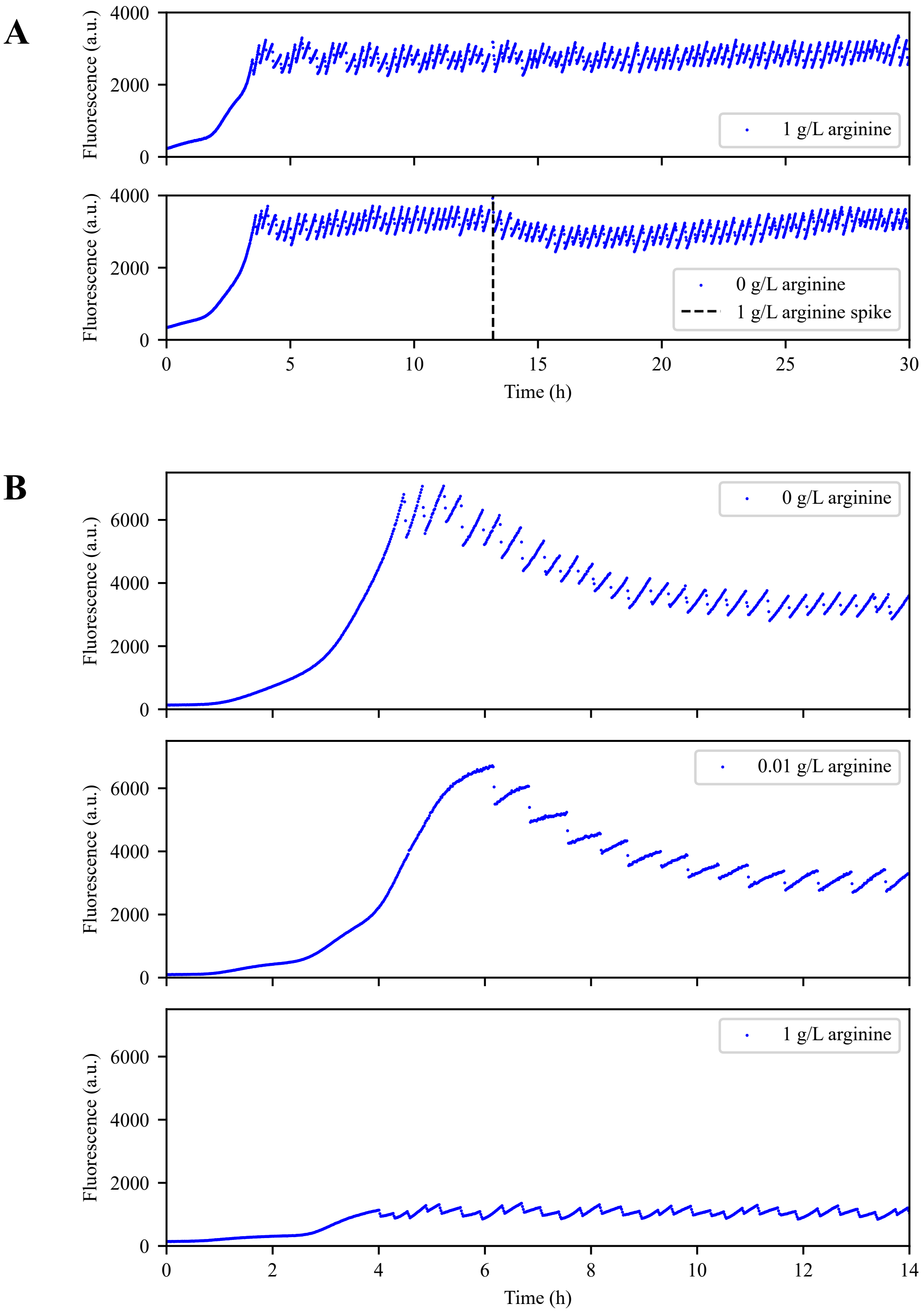
A
Fluorescence (a.u.)
1 g/L arginine
0 g/L arginine
1 g/L arginine spike
Time (h)
B
0 g/L arginine
0.01 g/L arginine
1 g/L arginine
Time (h)

Figure S6: effect of arginine on pyoverdine production, related to Figure 3. (A) Effect of arginine with complex amino acid mixtures on oscillatory behaviour of pyoverdine production. *P. putida* monocultures grown with $0.2\,\%$ CAA and $1\,\mathrm{g\,L^{-1}}$ arginine (top) still has dampened oscillatory behaviour at $29\,°\mathrm{C}$. When grown with $0.2\,\%$ CAA but no arginine, and then spiked with $1\,\mathrm{g\,L^{-1}}$ arginine at around time = 13 hours (bottom), pyoverdine production drops briefly before rising slowly. **(B) Effect of arginine with simple amino acid mixtures on oscillatory behaviour of pyoverdine production.** *P. putida* monocultures grown with $3\,\mathrm{g\,L^{-1}}$ serine and $0.5\,\mathrm{g\,L^{-1}}$ glutamine, using values suggested from Maser et al.[1] along with a range of arginine concentrations. Arginine has a clear effect, with lower concentrations appearing to dampen oscillations but cause a peak in pyoverdine before settling. Excess arginine creates oscillations that did not dampen after 15 hours.

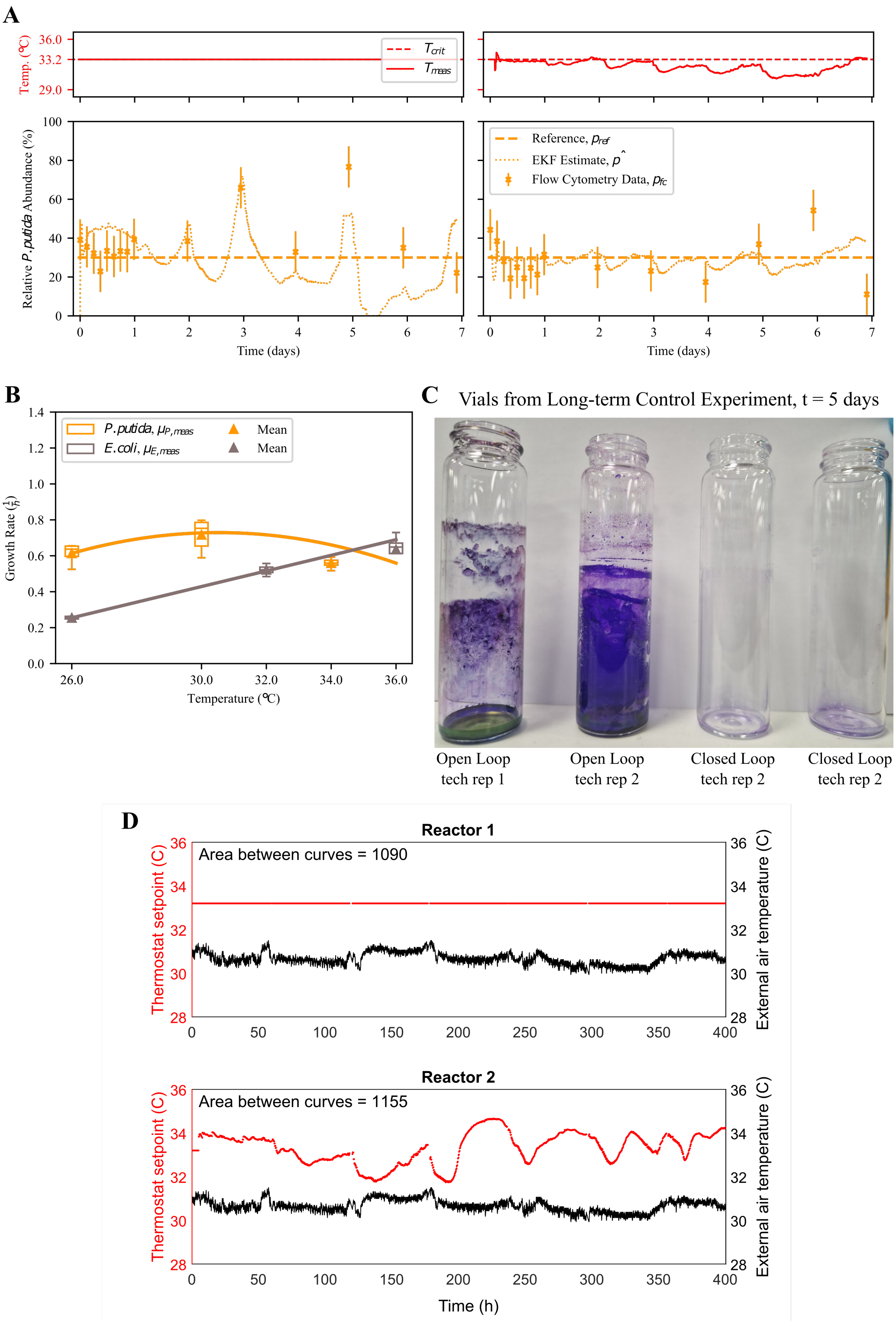
A
Temp. (℃)
36.0
33.2
29.0
T_{crit}
T_{meas}
Relative P. putida Abundance (%)
Reference, p_{ref}
EKF Estimate, $\hat{p}$
Flow Cytometry Data, p_{fc}
Time (days)
B
P. putida, $\mu_{P,meas}$
E. coli, $\mu_{E,meas}$
Mean
Mean
Growth Rate ($\frac{1}{h}$)
Temperature (℃)
C
Vials from Long-term Control Experiment, t = 5 days
Open Loop tech rep 1
Open Loop tech rep 2
Closed Loop tech rep 2
Closed Loop tech rep 2
D
Reactor 1
Area between curves = 1090
Thermostat setpoint (C)
External air temperature (C)
Reactor 2
Area between curves = 1155
Time (h)

Figure S7: long term co-culture control, related to Figure 7. (A) Additional replicates of long-term control. Similar trends are observed to replicates from Fig. 6, where open-loop control maintains the composition around the setpoint for a day, but then experiences large fluctuations over the rest of the week. The closed-loop control is still around the setpoint by the end of the experiment, though the *P. putida* abundance rose to around $50\,\%$ on the second last day. **(A) Effect of CAA concentration on growth rate.** Growth rate of monocultures cultured at a range of CAA concentrations. Because *E. coli* is much more affected by decreasing [CAA] than *P. putida*, the critical temperature is increased and there is a smaller temperature window to select for *E. coli*. The smaller difference in relative growth rate means that the system would respond slower to control inputs. **(C) Vials from the long-term co-culture control experiments, t=5 days.** Vials are stained with crystal violet, depth of purple represents amount of biofilm. Open-loop technical replicates had no controller, but the media temperature was set to the critical temperature. Closed-loop technical replicates uses the controller. Closed-loop vials have significantly less biofilm, presumably because the *P. putida* population was maintained at around $30\,\%$. **(D) Energy expenditure needed for control.** Temperature of the thermostat (i.e. temperature that the reactor maintained) in red for a reactor kept at a constant temperature (top) or a fluctuating temperature (bottom) for control to maintain a static composition from Fig. 7. The ambient temperature of the air outside the reactor is in black. The power used by the reactor to heat the culture is linearly proportional to the temperature difference, and hence total energy expended is (approximately) proportional to the area between the thermostat and external air temperature curves. In the reactor with feedback control, periods of heating above the setpoint would draw additional power, but periods of cooling below the setpoint draw none. The relative area between the curves is 1090 for a static setpoint and 1155 for feedback control, representing a 6% increase in energy expenditure to achieve compositional control.

References

1. Maser, A., Peebo, K., Vilu, R., and Nahku, R. (2020). Amino acids are key substrates to *Escherichia coli* BW25113 for achieving high specific growth rate. Research in Microbiology *171*, 185–193. URL: `https://www.sciencedirect.com/science/article/pii/S0923250820300176`. doi: 10.1016/j.resmic.2020.02.001.
