# Supplementary material for: Directing microbial co-culture composition using cybernetic control
Source: Cell Rep Methods. 2025 Mar 24;5(3):101009. doi: 10.1016/j.crmeth.2025.101009 (PMC12049730; doi:10.1016/j.crmeth.2025.101009)
Supplement: Document S2. Article plus supplemental information [file mmc2.pdf]

Cell Reports
Methods

Article

Directing microbial co-culture composition using cybernetic control

Graphical abstract

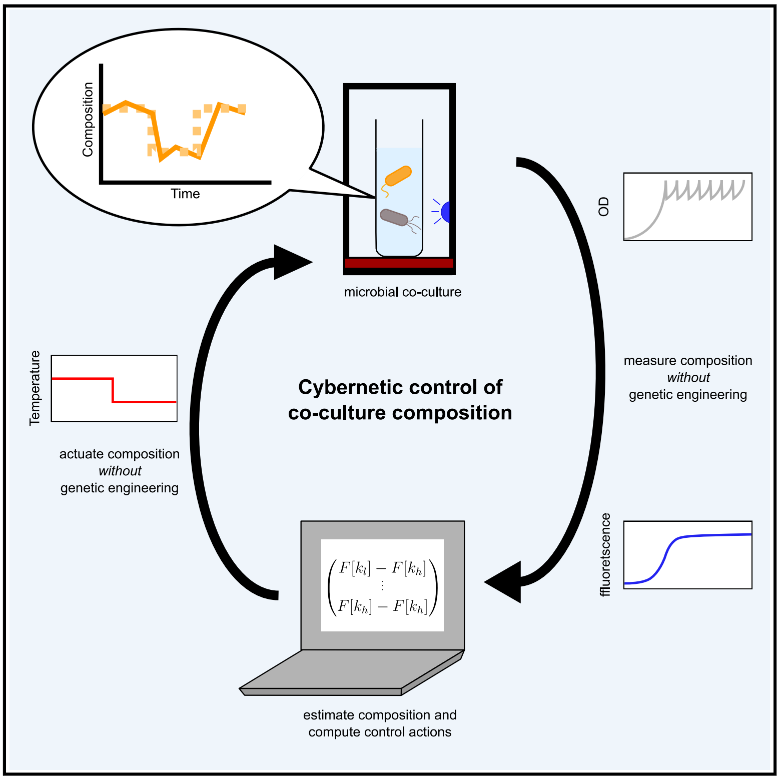

Highlights

- Computer-based control of the composition of a two-species bacterial co-culture
- Interfaced cells and computer without genetic engineering
- Combined bioreactor measurements and system model to estimate composition
- Adjusted temperature to actuate composition, achieving >1 week of dynamic control

Authors

Ting An Lee, Jan Morlock, John Allan, Harrison Steel

Correspondence

harrison.steel@eng.ox.ac.uk

In brief

The compositional instability of microbial co-cultures limits their potential in bioproduction. Computer-based cybernetic approaches enable dynamic control and real-time noise rejection. Lee et al. extend this method to be independent of genetic engineering, interfacing cells with computers by exploiting their natural characteristics, thus relieving burden and avoiding control-breaking escape mutations.

Lee et al., 2025, Cell Reports Methods *5*, 101009
March 24, 2025 © 2025 The Author(s). Published by Elsevier Inc.
https://doi.org/10.1016/j.crmeth.2025.101009

CellPress

Cell Reports Methods

CellPress
OPEN ACCESS

Article

Directing microbial co-culture composition using cybernetic control

Ting An Lee,[1] Jan Morlock,[2] John Allan,[1] and Harrison Steel[1,3,*]
[1]Department of Engineering Science, University of Oxford, Oxford OX1 3PJ, UK
[2]Department of Mechanical and Process Engineering, ETH Zurich, 8092 Zurich, Switzerland
[3]Lead contact
*Correspondence: harrison.steel@eng.ox.ac.uk
https://doi.org/10.1016/j.crmeth.2025.101009

MOTIVATION A key obstacle in the widespread application of microbial co-cultures in bioprocesses is their compositional instability. While several synthetic biology approaches have demonstrated control over co-culture composition, there has been an increased interest in computer-based cybernetic control approaches that can offload burdensome genetic control circuitry to computers and enable dynamic control and real-time noise rejection. This work extends that approach, demonstrating a cybernetic control method that is not reliant on any genetic engineering, instead interfacing cells with computers by exploiting their natural characteristics to measure and actuate the composition.

SUMMARY

We demonstrate a cybernetic approach to control the composition of a *P. putida* and *E. coli* co-culture that does not rely on genetic engineering to interface cells with computers. We first show how composition information can be extracted from different bioreactor measurements and then combined with a system model using an extended Kalman filter to generate accurate estimates of a noisy system. We then demonstrate that adjusting the culture temperature can drive the composition due to the species' different optimal temperatures. Using a proportional-integral control algorithm, we are able to track dynamic references with real-time noise rejection and independence from starting conditions such as inoculation ratio. We stabilize the co-culture for 7 days (∼250 generations) with the experiment ending before the cells could adapt out of the control. This cybernetic framework is broadly applicable, with different microbes' unique characteristics enabling robust control over diverse co-cultures.

INTRODUCTION

The use of microbial co-cultures for biotechnological applications has many potential advantages over monocultures: improved productivity from specialization or splitting of enzymatic pathways between species (division of labor),[1,2] the induction of valuable metabolites,[3] increased robustness to contamination,[4] or the ability to utilize cheaper substrates by combining strains with different capabilities.[5] A key obstacle preventing the widespread adoption of co-culturing methods is the challenge posed by engineering and controlling their composition.[6] In particular—and as stated by the competitive exclusion principle[7]—when multiple species compete for a limiting resource in a well-mixed co-culture, the faster-growing species will outcompete and eventually take over, reverting the system to a monoculture. Therefore, a major open challenge in the field is developing robust methods to control and stabilize co-culture composition. Such methods would enable broad applications of co-cultures and also accelerate their study and exploitation in general: the ability to dynamically control co-cultures allows a scientist to quickly explore the composition parameter space and thus maximize bioprocess productivity[8,9] or probe interspecies dynamics.

To address this control challenge, many engineered biological control systems have been developed to regulate co-culture composition. This includes methods based on complementary auxotrophies,[10] quorum sensing circuits,[11] burden growth rate actuators,[12] and more.[13] While such methods have proven effective for some applications, they have a number of limitations. One is that methods that require genetic engineering of constituent microbes are difficult to implement in complex communities that include non-model organisms and may encounter regulatory challenges if a co-culture is deployed in applications beyond the laboratory. Second is the metabolic burden imparted by genetically encoded parts, which manifests both as the diversion of cellular resources from bioproduction toward governing composition—potentially decreasing productivity—as well as an increased selective pressure against the controller,

This is an open access article under the CC BY license (http://creativecommons.org/licenses/by/4.0/).

encouraging escape mutations that break the control circuitry. Both of these limitations are exacerbated if attempting more advanced control with more complex (and thus more burdensome) genetic circuitry.

To mitigate the above challenges, computer-based cybernetic approaches[14,15] have been used to control composition by shifting some or all of the composition control functions from biological circuitry to computers.[16–19] These control methods make real-time estimates of co-culture composition, compare this measurement to a desired reference composition, compute a control action predicted to drive composition closer to the reference, and then execute these actions. Strengths of cybernetic approaches include noise rejection, the ability to account for uncertain measurements, and the ability to track dynamic references that benefit processes where the optimal composition changes over time.[14,20] Past realizations of cybernetic co-culture control systems have used computers to calculate control actions. However, these actions have typically been implemented by genetic engineering of co-culture members to establish suitable computer-cell interfaces. These may include optical[18] or chemical[21] responsive systems introduced to co-culture members to actuate changes in composition and typically relied on fluorescent reporters that are measured as a proxy for species abundance.[16–18,21] Such engineered biological interfaces allow (comparatively) straightforward implementation of computer-implemented control algorithms; however, control performance is conditional on the continued functioning of the genetically engineered interfaces. This can lead to undesirable fragility—genetically encoded parts (which are not part of the host cell's core functionality) are often perturbed by mutations, which can change/eliminate their function and hence the co-culture's behavior from the controller's actions. For example, Gutiérrez et al.[18] demonstrated excellent control of a two-strain *Escherichia coli* (*E. coli*) co-culture for 40 h, but after this point, escape mutations led to drift despite the computational control component's attempt to compensate.

We propose that by exploiting natural microbial characteristics and combining several population-averaged measurements, co-culture composition can be controlled with a cybernetic approach that does not require genetically encoded systems for measuring or actuating the composition. For a given two-strain co-culture (Figure 1A), this first requires identifying suitable microbial characteristics that might be used to actuate the growth rates, which could be any environmental condition where the species' optima do not completely overlap. By altering this condition, we can thereby differentially favor the growth of one strain over the other. Culture composition can be determined through projected growth rates under different conditions (i.e., from just optical density [OD] measurements), with improved accuracy if the strains have any other measurable characteristic that distinguishes them (e.g., a natural fluorophore). Once identified, such a system can be characterized in monocultures to parameterize the relationship between growth rates (or other measured parameters) and chosen control inputs. After testing this relationship in a co-culture, this can then be used to parameterize a mathematical model and design a computer controller. Finally, these can be combined to implement cybernetic control in a co-culture, and the results can be used to iteratively improve the model and controller if desired.

In this paper, we realize such an approach by demonstrating long-term dynamic control over the composition of a *Pseudomonas putida* (*P. putida*) and *E. coli* co-culture. This is achieved by combining simple control algorithms with actuation via small temperature changes and estimation of co-culture composition by combining measurements of optical density and fluorescence and their variation in time. We initially parameterize growth and production models for each co-culture member in monoculture experiments, then evaluate and adjust their behavior in co-cultures. In the next step, we apply the model with minimal adjustments to enable control in a co-culture setting, demonstrating its strong performance in over 25 independent bioreactor co-culture experiments. Key innovations of our work include our model-based approach to translating measurements performed on monocultures to derive accurate models of a mixed co-culture's behavior, methodology for dynamic estimation of composition for a co-culture based on multiple *in situ* population-averaged measurements of its behavior (Figure 1B), and demonstration that, when combined, these approaches can be used to dynamically control a co-culture composition or stabilize its makeup for 7 days ($\sim$ 250 generations), with the experiment ending before escape mutations overcome control.

RESULTS

We implemented cybernetic control over the composition of a 20 mL *P. putida* and *E. coli* co-culture grown in a Chi.Bio,[22] a bioreactor with heating, liquid handling, and fluorescence spectrometry capabilities. To achieve this control, we first identified and characterized their growth rate, dynamics, and fluorescent output (for *P. putida*) over different conditions. We then showed that small adjustments can actuate changes in co-culture composition. Combining the two species into co-cultures grown at low or high temperatures can favor one species over the other as expected but with undesirable behaviors that hinder robust control. After overcoming these challenges, we used the experimental data to build a system model and state estimator that together are used to demonstrate control.

We initially used the *P. putida* KT2440 strain in our experiments but later swapped to a *P. putida* OUS82-derived strain to improve estimation and control. This strain has a *lapA* gene knockout, which reduces its ability to attach to surfaces and form biofilms.[23,24] All results presented in this work (i.e., characterization, control experiments) that mention *P. putida* refer to and are derived from OUS82, except for those in the "challenges in co-culture implementation" section, which specifically highlight the issues with KT2440.

Measuring co-culture composition

We began by identifying properties of our *P. putida* and *E. coli* co-culture that could be exploited to estimate its composition. With a view toward bioprocess applications where integrating flow cytometers or microscopes with bioreactors may be costly or impractical, we instead looked toward bulk culture OD and fluorescence measurements, which bioreactors can measure *in situ*. The Chi.Bio bioreactor[22] measures OD at 650 nm every

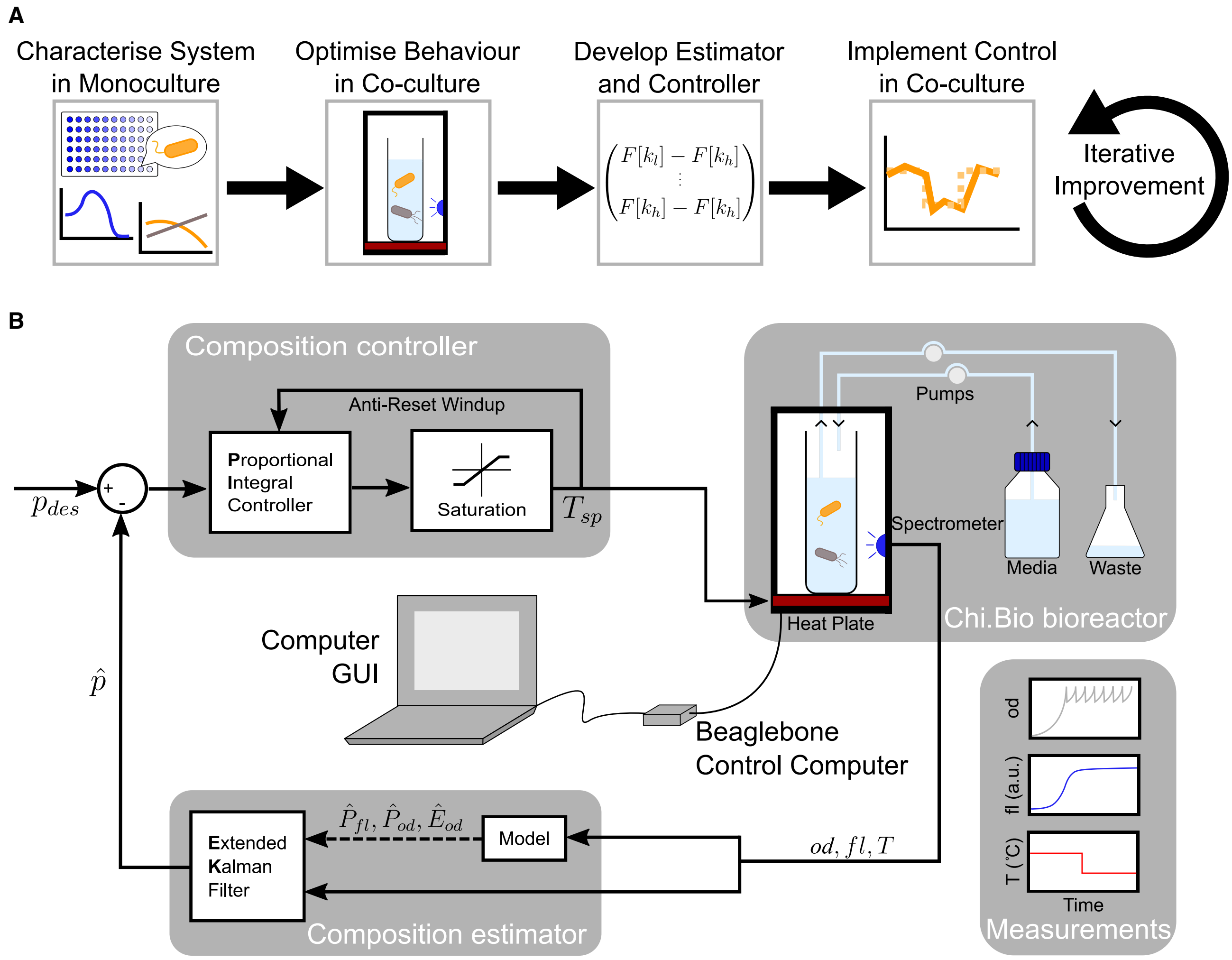

Figure 1. Cybernetic control approach and implementation

(A) Process of implementing cybernetic control of a co-culture. Begin by identifying suitable inputs and outputs to the system in monocultures, then combine both species and optimize the co-culture setup for better observability and controllability of the composition. Derive and parameterize a composition estimator and controller, then integrate them into the experimental setup. Finally, iteratively improve the setup, model, and control in co-culture experiments.

(B) Automated cybernetic control of a *P. putida* and *E. coli* co-culture. The bacteria are cultured in Chi.Bio bioreactors functioning as turbidostats, where pumps dilute with fresh media and pump out waste. Spectrometers and temperature sensors take online measurements of bulk culture OD (*od*), fluorescence (*fl*) and media temperature (*T*) every minute, then feed these measurements into the control computer where a model of the system and an EKF derives an estimate of relative *P. putida* abundance ($\hat{p}$). The difference between $\hat{p}$ and desired *P. putida* abundance (p_{des}) is used by a PI controller to calculate a culture temperature setpoint (T_{sp}) that would drive the co-culture toward the desired composition. A saturation block prevents unsuitable temperature setpoints, and an anti-reset windup decreases integral buildup. The bioreactors' heatplates alter culture temperature to the new T_{sp}, affecting *P. putida* and *E. coli* growth rates and thus composition, and the process is repeated the next minute.

minute and maintains the co-culture at an OD setpoint (i.e., functioning as a turbidostat) by routinely diluting the culture with fresh media when it rises above a setpoint. The OD provides information about the co-culture composition in how it changes over time (see "state estimation" section).

To acquire more information on the composition, we identified differential fluorescence behaviors of both strains (as stationary phase monocultures) via a 2D fluorescence scan in a plate reader (Figure 2A). This highlighted an emission peak for *P. putida* at ~460 nm when excited at ~410 nm, which is characteristic of the fluorescence of pyoverdine, an iron-chelating siderophore produced by the *Psuedomonas* genus.[25] *E. coli* had negligible observable fluorescence in this region, though some response is observed at excitation/emission wavelengths of 420/500 nm. As the Chi.Bio can measure this fluorescence signal by exciting the co-culture at 395 nm and measuring emissions at 440 nm, we showed that *P. putida* was producing a fluorescent molecule (assumed to be pyoverdine) at a concentration sufficient to be observed and could derive information on the amount of *P. putida* in the co-culture from its absolute value and dynamics.

Compared to constitutively expressed engineered fluorescent reporters, naturally produced fluorescent molecules may exhibit

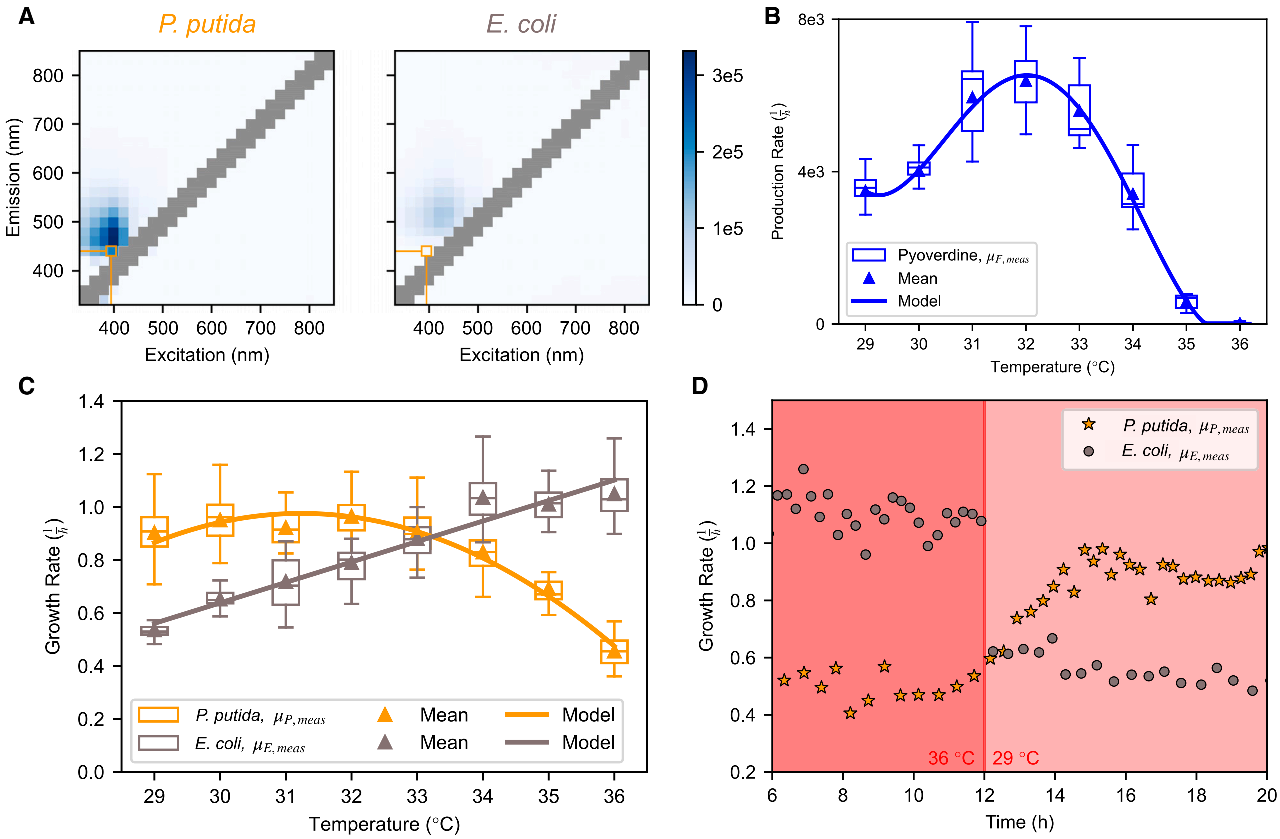

Figure 2. Fluorescence characteristics and temperature response

(A) 2D excitation-emission fluorescence scan of *P. putida* (left) and *E. coli* (right) grown to stationary phase in a 96-well plate. *P. putida* naturally produces pyoverdine, a fluorescent siderophore with peak emission around 460 nm when excited around 410 nm. The wavelengths used by the Chi.Bio in this work are 395/440 nm for excitation/emission (orange box).

(B) Production rate of pyoverdine (h^{-1}) at different temperatures peaking at 32°C, calculated by measuring fluorescence of a *P. putida* monoculture. The fourth-order polynomial fit is calculated using the mean value at each temperature.

(C) Growth rate of *P. putida* and *E. coli* (h^{-1}) at temperatures between 29°C and 36°C. A second-order (*P. putida*) and first-order (*E. coli*) polynomial fit is calculated using the mean value at each temperature.

(D) Growth rate dynamics of *P. putida* and *E. coli* calculated from monocultures, where dark red background = 36°C and light red background = 29°C. While the *E. coli* growth immediately responds to the temperature change by dropping from 1.1 h^{-1} to 0.6 h^{-1}, *P. putida*'s growth rate responds gradually, only reaching 1.0 h^{-1} after about 2.5 h.

complex behaviors that depend on the host cell's state. Using such a reporter as a measurement tool therefore requires characterization of its dynamics under the range of conditions anticipated during experiments—which here meant understanding how pyoverdine production varied as a function of temperature. We did this by estimating pyoverdine production from the fluorescence of *P. putida* monocultures grown at different temperatures, accounting for how different growth rates affected the dilution of pyoverdine molecules, and observed that production peaks near 32°C and decreases at temperature extremes. This temperature-pyoverdine relationship provides an additional parameter that can be used to infer *P. putida* abundance from current fluorescence values and historic temperature setpoints.

To ensure that fluorescence readings were comparable between reactors, calibration parameters for each reactor were measured representing a reactor's characteristic fluorescence offset and scaling factor, respectively (Figure S1A), which also confirmed that fluorescence scaled linearly with pyoverdine concentration within the range relevant to our experiments. We also showed that temperature had no significant effect on the fluorescence of pyoverdine in media with no cells (Figure S1B), supporting our hypothesis that changes in observed fluorescence are due to increased pyoverdine production rather than temperature dependence of measured fluorescence from a fixed quantity of pyoverdine. We also present properties of the fluorescence measurements taken by the Chi.Bio in Figures S1C–S1E, illustrating that the overlap, even when accounting for the hardware's excitation/emission spectra, is minimal.

Finally, to independently measure a "ground truth" co-culture composition during control experiments, an alternative offline measurement was required. This was initially attempted by sampling from the co-culture and plating diluted samples onto chloramphenicol and antibiotic-free plates, as *P. putida* is naturally resistant to chloramphenicol (*E. coli* estimated as the total

number of colonies − the number of *P. putida* colonies). However, while this could show changes in orders of magnitude, even after some optimization, it remained too variable to allow discrimination of small composition changes (Figures S3C and S3D). To address this, we employed an *E. coli* strain that contains an mKate2 (red fluorescent protein [RFP]) cassette, used only for validating control in flow cytometry after bioreactor runs have already concluded. This was necessary because samples were stored at −80°C, and we observed that pyoverdine's fluorescent signal was lost after a freeze thaw (Figure S2C), which prevented consistent flow cytometric gating and was hypothesized to be due to pyoverdine leaking out of cells. The fluorescent reporter is not used at any point for control of the co-culture and is too weakly expressed (PRNA1 promoter) for any signal to be observed in a Chi.Bio when excited at its peak wavelength.

Actuating change in co-culture composition

Once composition could be measured, we needed a way to adjust co-culture composition to direct it toward desired reference compositions. Possible methods include tuning the concentration of chloramphenicol (increase to favor *P. putida* and vice versa) or a carbon source that only one species can utilize (e.g., lactose, only metabolized by *E. coli*), but for ease of implementation and broad applicability, we chose to exploit their different temperature optima. Previous work[26] has already shown that temperature can be used to influence the final composition of a batch culture of these species as *P. putida* grows optimally at a lower temperature than *E. coli*.

To characterize the relationship between temperature and growth rate under our experimental conditions (Figure 2C), *E. coli* and *P. putida* were grown in monocultures at a set temperature for 12 h. The reactors act as a turbidostat (continuously diluting the culture) that "dithers" the OD from 0.44 to 0.56 (Figure S3A), i.e., repeatedly allowing the OD to increase until 0.56 before diluting it down to 0.44 with fresh media. Afterward, the temperature is swapped to a different setpoint for 12 h to observe the growth response to a change in temperature. *P. putida* grows quickest at around 31°C and slower at either temperature extreme, while *E. coli*'s growth rate increases linearly with temperature. At around 33.2°C, their growth rates intersect, yielding a temperature setpoint where theoretically a co-culture of any composition could be maintained. In contrast, temperature setpoints above or below this critical point favor the growth of *E. coli* or *P. putida*, respectively. Temperature increases are rapid with the Chi.Bio's heatplate actively heating the culture, while temperature drops are slower, relying only on passive cooling (timescales dependent on the magnitude of change).

While investigating the time dependence of our growth-temperature relationship, we observed the two species respond with different timescales when the temperature changes: the *E. coli* growth rate drops sharply when the temperature changes from 29°C to 36°C (Figure 2D), while the *P. putida* growth rate gradually decreases for around 2.5 h before settling. The same dynamics are observed (Figure S3B) when the temperature transition is reversed (i.e., cold to hot), indicating that this lag is specific to the species and not the direction of temperature change.

Finally, our characterization approach implicitly assumes the temperature-growth relationship in monoculture will be similar to that in co-culture—this was not obvious *a priori* as factors such as density dependence or inter-species interactions could lead to different dynamics in co-culture. Nevertheless, subsequent sections show this assumption was adequate for our application, though this is aided by the use of closed-loop feedback, which reduces sensitivity to system parameters.

Challenges in co-culture implementation

After initial characterization in monoculture, we investigated the effect of temperature on a co-culture. This was initially done with the *P. putida* KT2440 strain. Monocultures were mixed and grown at either 26°C or 37°C (Figure 3A)—as expected, 26°C strongly favored *P. putida* KT2440 with no *E. coli* observed after less than 4 h. However, this experiment demonstrated that despite *E. coli*'s significant growth rate advantage at 37°C, *P. putida* KT2440 persists in the co-culture and, after an initial fall in abundance, even seems to gradually increase over time.

Upon further investigation, we observed a biofilm concentrated around the air-water interface that grew in co-cultures and *P. putida* KT2440 monocultures (especially visible when stained with 0.1% crystal violet, Figure 3B) but not *E. coli* monocultures. This suggests that *P. putida* KT2440 causes a biofilm to form in the reactor in either case, and Figure S3E illustrates that while the co-culture biofilm contains both species, it consists primarily of *P. putida*. The biofilms are not particularly thick around the bottom third of the tube where the OD and fluorescence measurements are taken. Additionally, transferring the culture to a fresh vial without biofilms did not significantly impact measurements (Figure 3C middle). Hence, we assumed that the biofilm was not significantly influencing the co-culture measurement through aberrant readings. Instead, we hypothesized that forming a biofilm granted *P. putida* KT2440 a strong selective advantage in the turbidostat environment as they remain in the bioreactor while planktonic cells are constantly pumped out of the culture. These biofilm cells would be able to divide without getting diluted but can be shed into solution due to natural detachment and sloughing,[27] aided by the shear forces from constant vigorous stirring, artificially increasing the observed growth rate. This ability of biofilm-forming cells in continuous cultures of a similar scale to adhere (escaping dilution) yet detach has been an observable and problematic selective advantage in automated lab evolution experiments,[28,29] necessitating the development of specialized hardware to overcome this.[30,31] As the biofilm is primarily *P. putida*, its net contribution is to increase the growth rate of *P. putida* relative to *E. coli*, thus allowing it to persist at temperatures where it should be quickly outcompeted by *E. coli*. We tested this by comparing a co-culture grown at 37°C with no vial changes (Figure 3C left), with a co-culture swapped into a fresh vial when fluorescence began to increase (i.e., when a biofilm had been established, Figure 3C middle), and with a co-culture swapped into a fresh vial every 4 h (i.e., before a biofilm had been observed to form, Figure 3C right). We observed that after the initial post-mix drop in fluorescence and *P. putida* KT2440 abundance, both fluorescence and abundance began to increase in the reactor with no fresh vial. Meanwhile, swapping into a fresh vial when fluorescence began to

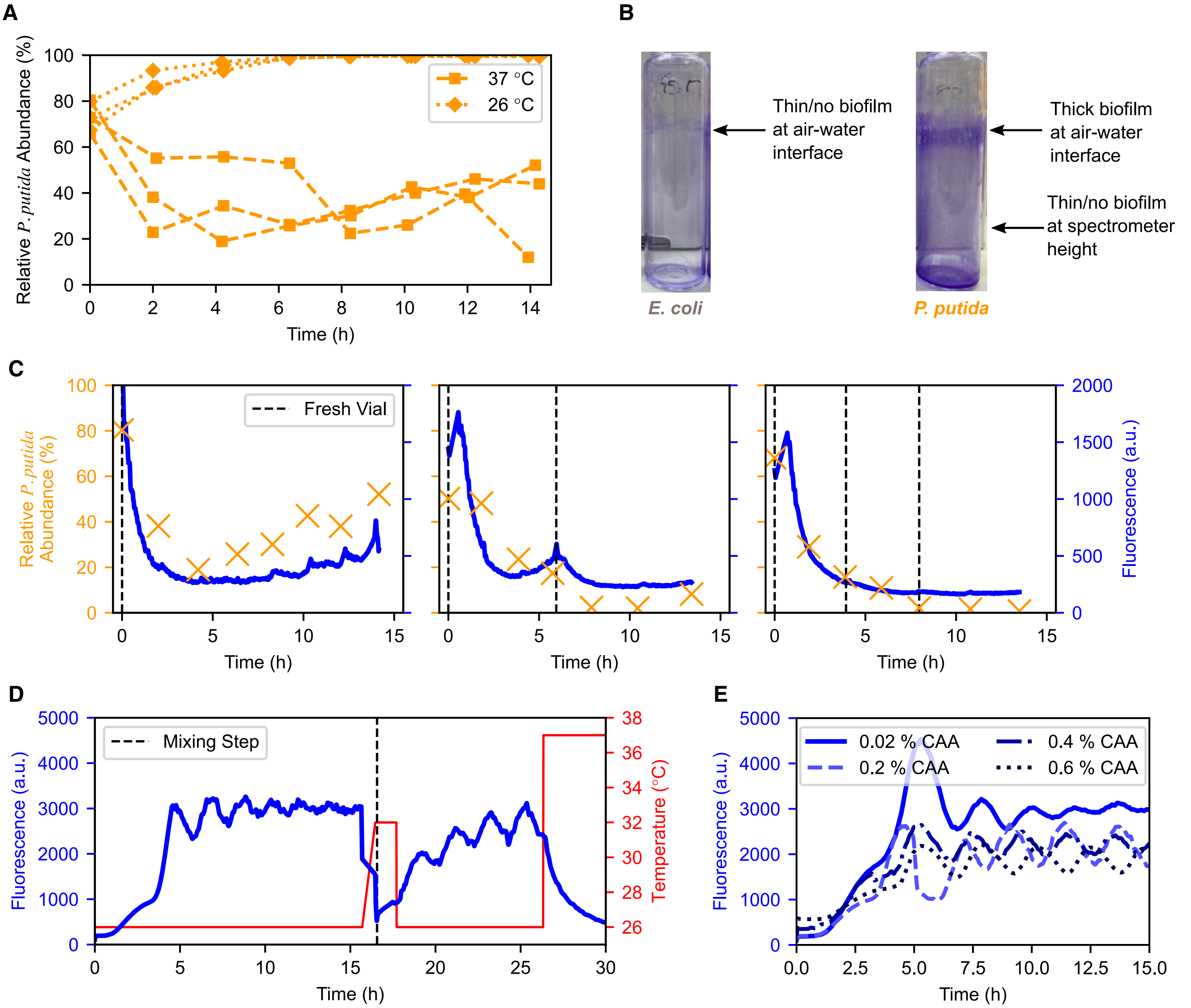

Figure 3. Challenges in co-culture implementation

(A) Relative abundance (%) of *P. putida* KT2440 and *E. coli* after mixing at $t = 0$ into a co-culture. At 26°C, *P. putida* KT2440 quickly and completely takes over the culture, while at 37°C, *E. coli* is favored, but *P. putida* KT2440 persists even after 14 h

(B) Biofilms stained with 0.1% crystal violet from *E. coli* (left) and *P. putida* KT2440 (right) overnight monocultures. The dark purple near the air-water interface of the *P. putida* KT2440 vial indicates thick biofilm, while there is little/no biofilm at spectrometer height for both.

(C) Relative *P. putida* KT2440 abundance (%) and fluorescence of co-cultures mixed at $t = 0$ and held at 37°C. Left: Without changing vials, biofilms consisting primarily of *P. putida* KT2440 (Figure S3E) slowly accumulate, artificially inflating *P. putida* growth and abundance, which in turn increases fluorescence. Center: Replacing the vial when fluorescence increased after about 6 h was sufficient to remove most *P. putida* KT2440 from the co-culture. The gradual rather than sharp decrease in fluorescence in the fresh vial indicates that the rising fluorescence was from a genuine increase in pyoverdine rather than biofilms obscuring the spectrometer. Right: By replacing vials every 4 h (i.e., before biofilms can form), the *P. putida* KT2440 is completely lost from the co-culture, as expected by its lower growth rate at 37°C.

(D) Fluorescence (a.u.) and temperature (°C) of a *P. putida* OUS82 monoculture, mixed 50:50 with an *E. coli* monoculture at ~16 h. At 26°C, the monoculture fluorescence first increases to around 3,000 a.u. as the culture reaches the OD setpoint (not shown) and then oscillates while slowly dampening over time. Upon mixing, fluorescence drops sharply by half (as half of the *P. putida* is replaced with *E. coli*) and continues to decrease as the temperature is set to an intermediate temperature of 32°C. When the temperature is reduced to 26°C, fluorescence rises back toward 3,000 a.u. while oscillating.

(E) Fluorescence (a.u.) of *P. putida* OUS82 monocultures over time when grown in M9 with a range of CAA concentrations at 26°C. Oscillations are present at all concentrations, but although 0.02% has a large spike upon reaching the OD setpoint, oscillations dampen over time, and at all other concentrations, they persist at the same amplitude over 15 h.

increase could reverse the trend, and swapping every 4 h prevented either fluorescence or abundance from recovering.

Attempting to mitigate short-term biofilm formation, we initially tested several anti-biofilm measures reported in the literature including Polysorbate 20 (Tween)[32] and cellulase.[33] While some were effective in standard biofilm assays in 96-well plates, they tended to be ineffective for cultures in Chi.Bio reactors, highlighting how different methods of culturing can create different phenotypes (Figures S6 and S7). This could be due to the overall cell density (cells reach a dense stationary phase in 96-well plates, while they are held in an early exponential phase in the Chi.Bio) or the fact that constant dilution in turbidostats selects for cells in biofilm regardless of chemical/biological intervention. We therefore changed from *P. putida* KT2440 to instead use *P. putida* OUS82 with a *lapA* gene knockout (the characterization data shown in the above sections are for the OUS82 strain, and it is used for all subsequent co-culture experiments). *lapA* encodes a surface adhesin described as "essential" for biofilm formation,[23,24] but even so, biofilms still appear after $\sim$ 24 h in *P. putida* OUS82 monocultures (or longer in co-cultures, depending on the population ratio). Because of this, cultures were transferred into sterilized, fresh tubes every 24 h.

While the above mitigations minimized the impact of biofilms on co-culture dynamics, parameterization of OUS82 revealed unexpected fluorescence dynamics unseen in KT2440: robust oscillations in pyoverdine production emerged that were highly consistent across replicates and persisted for days even after mixing in co-culture (Figure 3D). These oscillations have a significant impact on the ability to estimate *P. putida* abundance and thus on control, as an increase in *P. putida* population is indistinguishable from the rising edge of an oscillation. Further experiments (Figures S5A–S5D) showed these oscillations were affected by temperature, becoming more prevalent at lower temperatures. This makes accurate calculation of the mean pyoverdine production rate challenging unless the entirety of an oscillation period had a constant temperature. The oscillations were also influenced by the concentration of casamino acid (CAA) (Figure S5E), particularly arginine (Figure S6B), which is known to be related to pyoverdine production.[34] The effect of several synthetic amino acid mixtures, nitrogen sources, and arginine concentrations either in media or spiked-in were tested (Figures S5 and S6) but could not completely remove oscillations at 26°C.

Ultimately, the simplest set of conditions that produced consistent results was 0.02% CAA and a minimum temperature of 29°C. Reducing CAA concentration dampened oscillations quicker (Figure 3E) at all temperatures, and staying above the minimum temperature prevented excessive oscillations. Static references were also less affected, as they depend on reaching a particular composition and then maintaining it with a 33.2°C critical point where few to no oscillations are observed. Removing CAA entirely was not practical because *E. coli* grows too slowly in comparison to *P. putida* without it (Figure S7B). The limited temperature range of 29°C– to 36°C was therefore chosen for actuation of the co-culture to trade off different behaviors of the combined system: at lower temperatures, the larger difference in growth rate (and quicker control response) would be offset by the larger oscillations, making composition estimation difficult. On the other hand, higher temperatures inhibit pyoverdine production (Figure 1B), again reducing our ability to accurately track composition.

State estimation and model

The complex dynamics of pyoverdine production (i.e., temperature dependence, oscillations) make bulk culture measurements of absolute fluorescence by itself an inaccurate direct proxy for *P. putida* abundance. A simple example is that a co-culture that is 99% *P. putida* but held at a high temperature for several hours will have almost no measurable fluorescence, despite consisting primarily of *P. putida*. To overcome this challenge, we combine several estimations derived from easily available bioreactor measurements to achieve an accurate estimation of composition:

(1) Absolute OD value: the combined abundance of both species
(2) Time derivative of OD: co-culture growth rate over time
(3) Absolute fluorescence value: the abundance of pyoverdine
(4) Time derivative of fluorescence: the production of pyoverdine over time

The absolute OD value (1) reflects the total abundance of cells. This requires calibration (e.g., against colony-forming unit [CFU] or flow cytometry) if a measure of the number of cells for each species is required (due to differences in size and absorption between species leading to different numerical densities for a given optical density). The cultures are grown in turbidostat mode (i.e., maintained around a set density) and so are periodically diluted when the absolute OD exceeds its setpoint, creating phases of dilution and phases of growth (Figure S3A)—we refer to each such period between dilution events as a growth period. The time derivative of OD (2) is a composite measure of the two species' growth rates over the last growth period. Since the temperature at every time point is known, as is the individual species' growth-temperature relationship, the time derivative of OD can provide the abundance estimates $\widehat{P}_{od}[k]$ and $\widehat{E}_{od}[k]$ (STAR Methods). As an example, a co-culture grown at 36°C with a high total co-culture growth rate (i.e., equal or similar to *E. coli* growth at that temperature) indicates a predominance of *E. coli*, while one with a low total co-culture growth rate should consist predominantly of *P. putida*. The absolute fluorescence values (3) provide the next source of information about composition through the relationship shown in Figure 2B, though as described above, this provides limited information in certain conditions. Finally, the time derivative of fluorescence (4) is the function of historical time-averaged *P. putida* abundance and the temperature and, given an understanding of the temperature-production relationship, can be used to infer the abundance estimate $\widehat{P}_{fl}[k]$ (STAR Methods).

Estimation method 1 reflects total density of the culture while estimation methods 2–4 provide an estimate of composition, each with distinct advantages and disadvantages (Table 1). To reduce the effect of measurement noise, methods based on time derivatives require multiple consecutive measurements. This results in a lower measurement update rate and reduces

Table 1. Qualitative comparison of estimation methods or co-culture composition

Measurement	Method	Update rate	Update Quality at Temperature			
			Change	Low	$\mu_E \approx \mu_P$	High
od	absolute	high	no information about composition			
	time derivative	intermediate	low	high	low	high
fl	absolute	high	high	low	high	low
	time derivative	intermediate	low	intermediate	high	low

their accuracy during periods in which temperature changes rapidly. More broadly, the quality of all estimation methods depends strongly on the magnitude and dynamics of culture temperature. For example, the quality of estimates from absolute fluorescence decreases at low temperatures when oscillations begin to appear, as well as at high temperatures where pyoverdine production is minimal (i.e., above 35°C, see Figure 2B). Curvature methods (particularly OD, which does not oscillate) remain effective at low and high temperatures; however, the accuracy of OD curvature measurement methods decreases whenever a culture is around the 33.2°C critical temperature, as (near) equivalent *P. putida* and *E. coli* growth rates mean the total co-culture growth rate becomes independent of composition. Together these comparisons highlight the complementary strengths and weaknesses of each estimation method, which when taken together, demonstrate that at least one method is viable in each condition encountered by our co-culture.

In order to combine each method to derive a single quantitative estimate of composition, which also accounts for each method's variable quality (and its dependence on the culture's current and past behavior), we employ an extended Kalman filter (EKF), a well-established control engineering tool that estimates the state of a system by fusing a system model with measurements and their uncertainty. Several works have explored how it can be used to improve observability of biological systems.[35–37] Here, we demonstrate it experimentally: every cycle (i.e., every minute), the abundance of *P. putida* $P[k]$, *E. coli* $E[k]$, and pyoverdine $F[k]$ are calculated in two steps, the prediction step and the measurement update step (Figure 4).

In the prediction step, the EKF uses an initial state estimate from the previous cycle, combining this with a system model to predict the current state of the system. The system model is described in detail in the STAR Methods; it uses differential equations to model the dynamics of each species' growth and pyoverdine production and is kept sufficiently simple (i.e., with a small number of variables and parameters) to facilitate system characterization. While this model could be used to make long-term forward predictions of the system state based only on an initial state estimate, any errors in the initial estimate or the model itself would be propagated forward, leading to divergence of the model from reality. Overcoming this divergence is the purpose of the measurement update step; here, the EKF updates the predicted state with the different measurements and intermediate estimates, weighted by their uncertainty so that low-quality updates (e.g., $\widehat{P}_{fl}[k]$ at a high temperature) contribute less to the final estimate. This allows the individual measurement update methods to complement each other and makes the resulting updated state estimate less prone to measurement and system model inaccuracies.

The capabilities and limitations of this approach are explored in Figure 5. A realistic simulation including plausible measurement noise and model mismatch (Figure 5A) demonstrates that while OD-based estimates $\widehat{P}_{od}[k]$ and $\widehat{E}_{od}[k]$ are helpful in the beginning, they were far away from the true composition toward the end of experiment when culture temperature was near to the critical temperature. Meanwhile, using only fluorescence in the measurement update step was insufficient when the temperature was at 36°C near the beginning but delivered good results afterward. Finally, we observe that the state estimate without any measurement update has no opportunity to correct for its model mismatch and (simulated) noise and continues to diverge over time. The result of this simulation is borne out in summary statistics from experimental co-culture control tests (Figure 5B), where the EKF with combined updates leads to more accurate composition estimates than the system model alone. This is observed in both short-term and long-term experiments, with the latter presenting a broader error distribution due to increased time for estimation error to build up. It is worth noting that due to biological and measurement variability during experiments, one anticipates cases in which the model-only approach is (by chance) closer to the actual density than the EKF output; nevertheless, the EKF approach outperforms model-only prediction at almost all time points in almost all experiments.

Control approach and implementation

The system's controller is tasked with setting the temperature such that the estimated relative *P. putida* abundance $\widehat{p}$ shifts toward the desired relative abundance p_{ref}. The family of proportional-integral-derivative (PID) controllers was chosen due to its computational simplicity and low model dependence.[38] The derivative term was not utilized as the proportional-integral (PI) controller demonstrated sufficient performance, and an overdamped (i.e., slow responding) control that introduces slower changes in temperature setpoints was found to improve state estimation. This fact also discouraged the use of a (computationally even simpler) bang-bang controller, which, given a dynamic system, usually results in zigzag-shaped temperature profiles. The output temperature was constrained to be within the 29°C to 36°C bounds. This constraint prevents large temperature fluctuations, preventing the controller from making swift adjustments that could otherwise lead to over/undershooting target community compositions but comes at the cost of possible "integrator wind-up" in the control.[38] An anti-windup reset was therefore added that decreases the integrated error once the temperature setpoint saturates. The control gains were initially

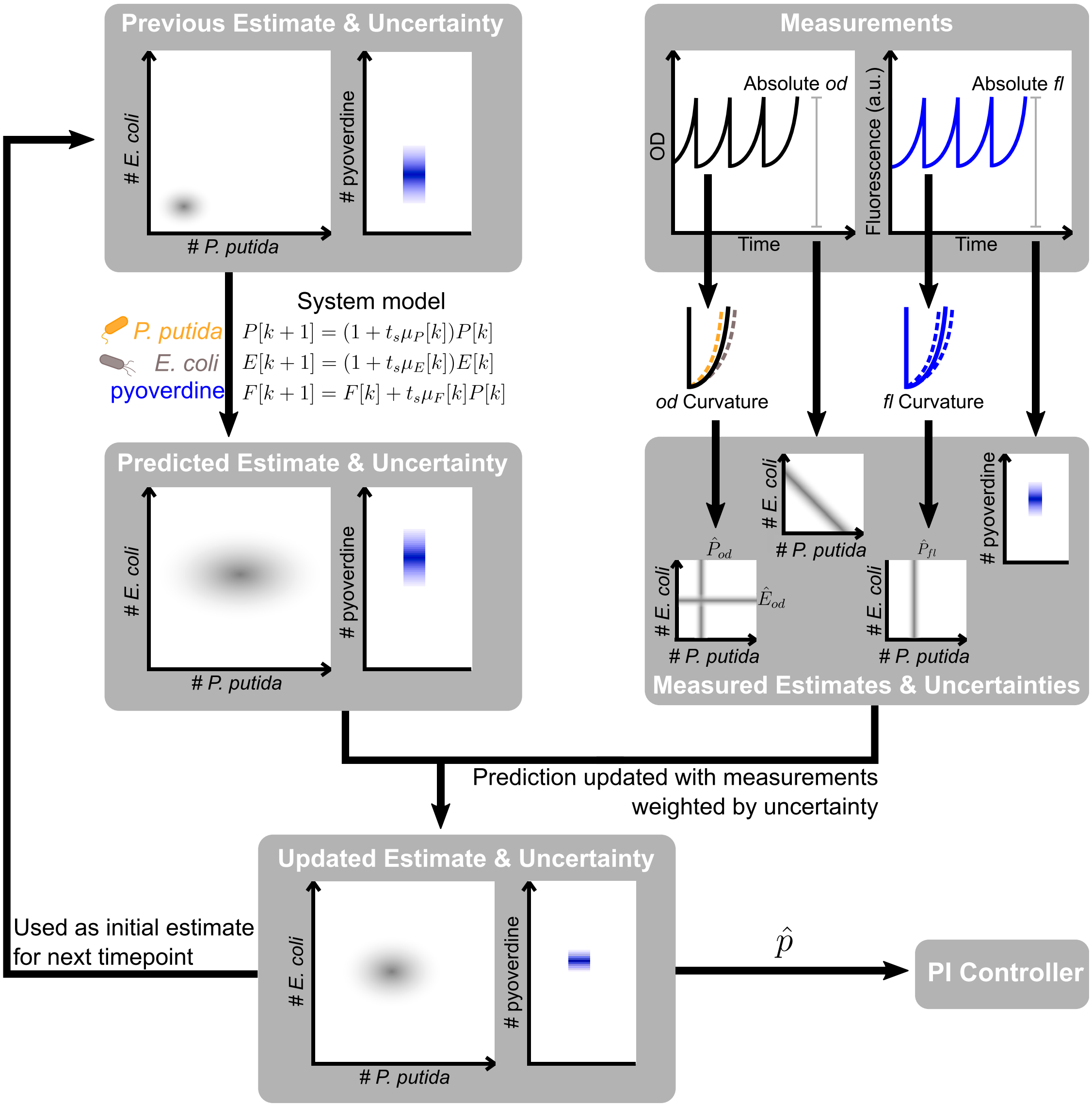

Figure 4. Extended Kalman filter for recursive online composition estimation

First, the current abundances of *P. putida*, *E. coli*, and pyoverdine are predicted based on the estimate from the previous cycle and the system model. In the next step, the bioreactor takes bulk culture fluorescence and OD measurements, whose curvature across a growth period is used to calculate the intermediate estimates $\hat{P}_{od}$, $\hat{E}_{od}$, and $\hat{P}_{fl}$. The EKF then updates the predicted abundances with the intermediate estimates and the absolute measurement values, taking into account the model and measurement uncertainties. Finally, from the resulting estimate, the relative abundance of *P. putida* ($\hat{p}$) is computed and used by the controller.

tuned in a simulation with final tuning being done in experiments. Both the EKF and controller were integrated into the Chi.Bio operating system in Python, running in real-time on the bioreactor's control computer (a Beaglebone Black microcontroller).

By combining measurement, state estimation, a control algorithm, and actuation, we are able to implement cybernetic control of the *P. putida*-*E. coli* co-culture. All control experiments had an initial 1-h period of growth at 33.2°C where the culture grew to reach the OD setpoint. We were able to demonstrate short-term static and dynamic reference tracking with a square wave pattern from 30% to 70% *P. putida* abundance over 1 day (Figure 6A). The controller first drives the composition to the target and then maintains it by staying around the critical temperature with only slight deviations. The estimation improves over time and is able to align with offline measurements within 5 h, negating the effect of variability in the initial inoculum mixing (i.e., when pre-cultures were combined to attain an approximately 50:50 population ratio), contrasting open-loop control, which would propagate errors through the rest of the experiment. Temporary estimator inaccuracies (such as initial offset from 50:50 co-culture) do not significantly impair the control due to the relatively slow dynamics of the system. The sudden

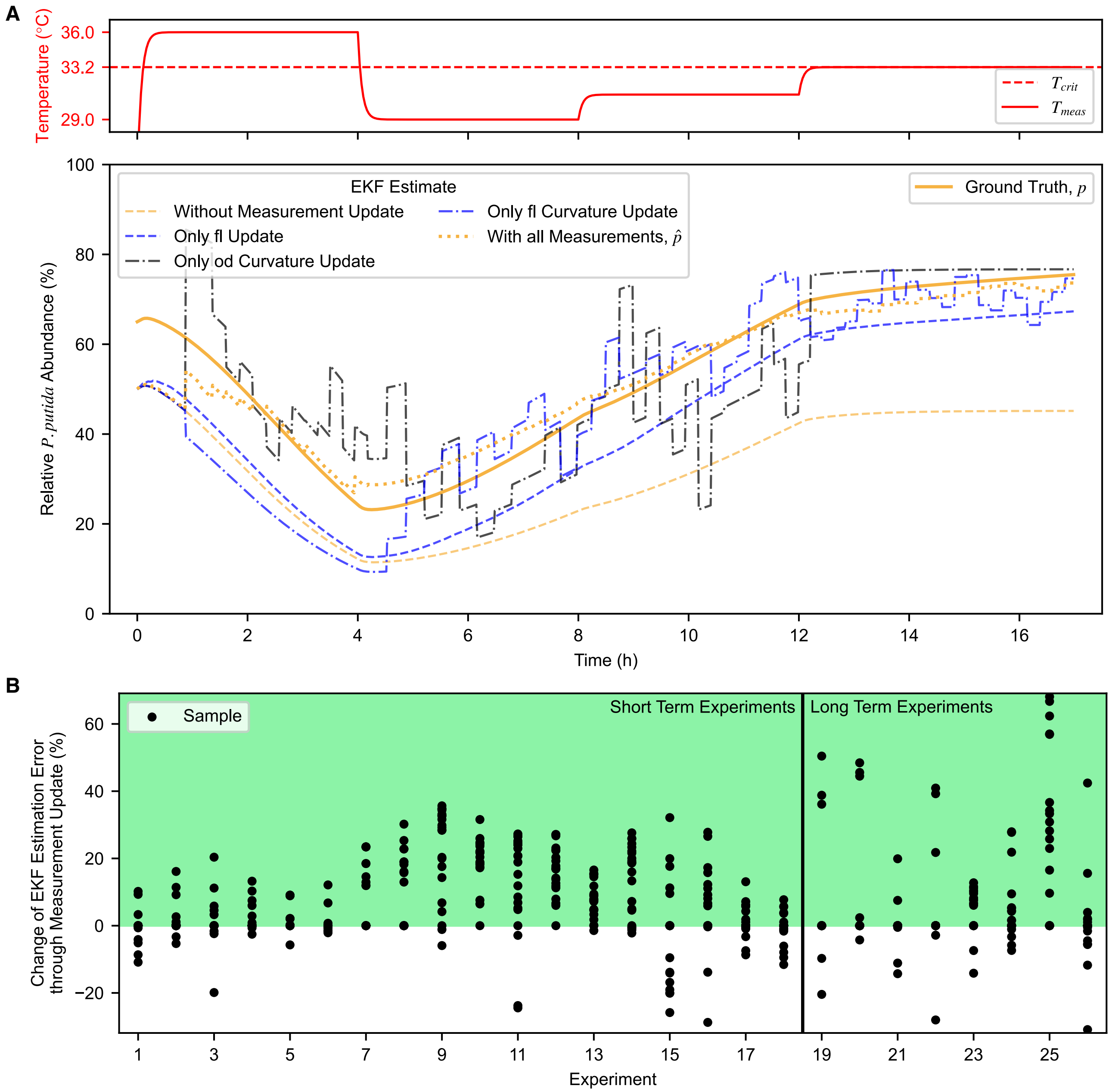

Figure 5. Comparison of different extended Kalman filter composition estimates

(A) Composition of a co-culture including plausible measurement noise and model mismatch, simulated using the characterized system model (labeled as ground truth, p). Top: The predefined temperature profile over 17 h. Bottom: The relative *P. putida* abundance estimates resulting from EKFs that employed either no measurement update, only a single update method, or all update methods combined.

(B) Comparison between the error of the relative *P. putida* estimate from the EKF $\hat{p}$ and the error of the estimate from the system model alone (i.e., without any measurement updates) with respect to the offline flow cytometry measurements. The data were collected across 26 real co-culture experiments. A value above zero (green) signifies the EKF with all measurement update methods outperforming (i.e., reducing error) compared to the model alone. Long-term experiments span multiple days, while short-term experiments ran for ~1 day.

drop around 16 h is assumed to be due to noise in the offline measurement process (storage/sample preparation/flow cytometry), as it is not consistent with adjacent time points given the temperature of the culture. We also demonstrate the response time of the controller with a sine wave reference (Figure 6B); here, composition responds with a delayed and damped sine wave of a similar period. This is primarily due to the integrator in the PI controller and the slow system dynamics that result in a gradual and delayed change in the composition after a change in the reference.

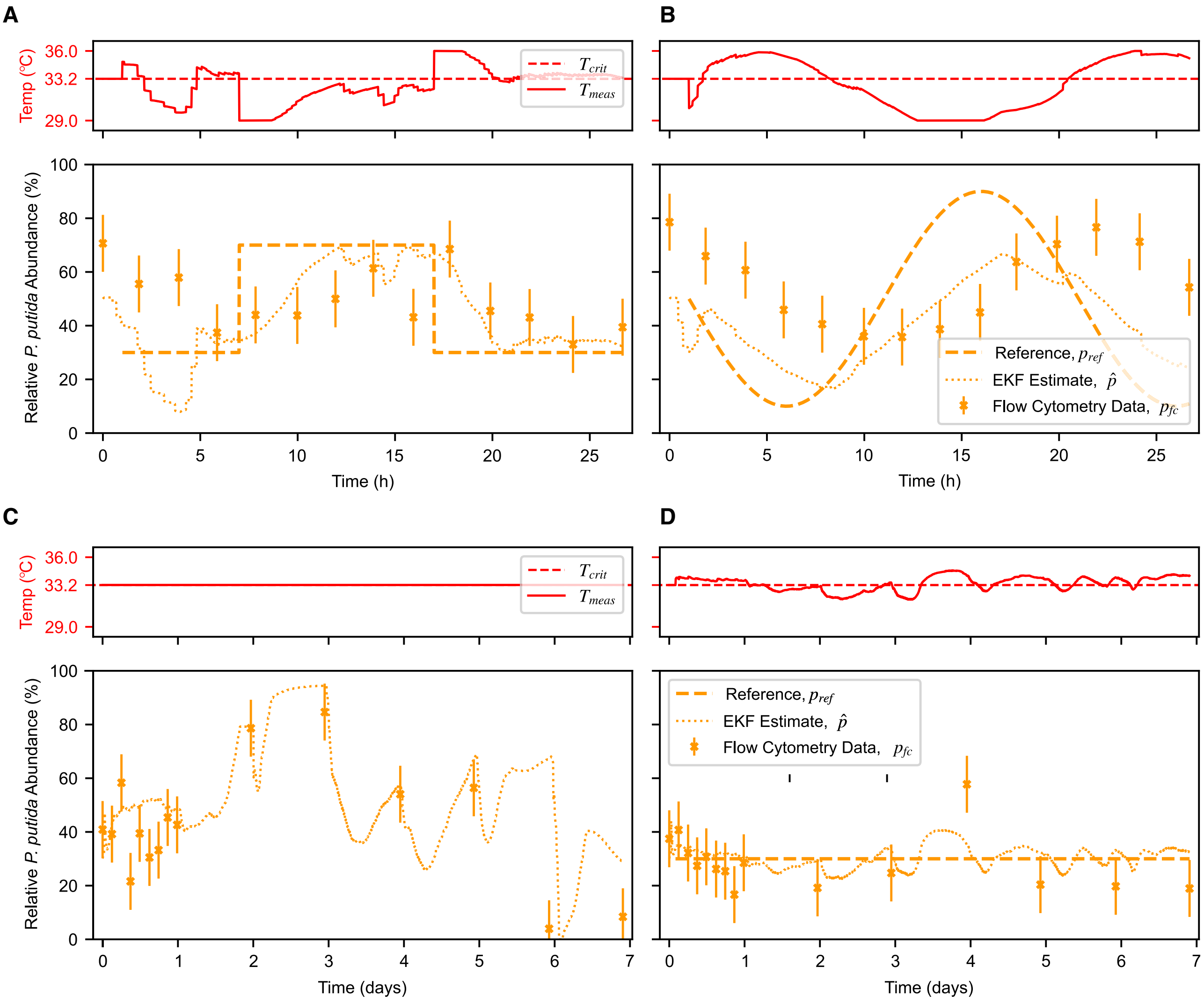

Figure 6. Open-loop and closed-loop control experiments

(A) Top: Media temperature over time, as set by the controller. Bottom: Relative *P. putida* abundance over time in a co-culture. Despite inaccuracy near the beginning (the estimator starts at an idealized 50:50 mix, but mixing is imperfect), the EKF estimate improves over time and the controller is able to track a square wave reference. Error bars are standard deviation derived from measured flow cytometry noise (Figure S2D).

(B) Controller with a sine wave reference. As expected, the simple PI controller cannot fully compensate for the slow system dynamics and causes a delayed response.

(C) Open-loop control of a co-culture over 1 week with starting ratio of 30:70 *P. putida*:*E. coli* grown at critical temperature 33.2°C, where theoretically the ratio would be maintained. Culture is swapped into fresh vials daily to prevent excessive biofilm formation, with samples for flow cytometry taken concurrently. While the composition is relatively static in the first 24 h, it subsequently varies significantly (from 5% to 85%). The EKF estimator tracks the composition well throughout the experiment.

(D) Closed-loop control with the same 30:70 reference. The controller was able to maintain the *P. putida* abundance at around 30% for the whole week, requiring slight deviations from the 33.2°C critical temperature in both directions.

While open-loop control of the co-culture could be possible given that the relationship between temperature and growth rate is known, without feedback, such an approach would be unable to respond to phenotypic variation or adaptation or indeed any genetic (evolutionary) adaptation that occurs during long-term growth experiments. For example, a co-culture mixed in a 30:70 ratio of *P. putida*:*E. coli* and held at the critical temperature should theoretically maintain that ratio as both species have an equivalent growth rate. This is investigated in Figure 6C, where the *P. putida* abundance fluctuated near 30% for the first 24 h, increased to ~80% after 2 days, and rose further before dropping to 10% after 7 days. Figure 6D illustrates a similar experiment, but now employing the PI feedback control system with a set-point of 30% for all 7 days. Excepting one outlier on day 4, the experiment shows that the setpoint was maintained throughout but with a consistent 10% offset between the online

estimated composition and the flow cytometric ground truth. This offset may be caused by the biofilms (of mixed composition) that formed in the vials even with daily vial replacement (Figure S7C): changes to the observed growth rate of either species (e.g., if it were to be artificially inflated by biofilm shedding cells into the media) lowers the quality of the OD curvature estimate. In addition to the possibility of systematic sampling noise in the flow cytometry protocol, flow cytometry only measures the composition of planktonic cells, completely ignoring biofilm cells that do contribute to EKF estimates as they both contribute to OD and fluorescence measurements.

Interestingly, while both biological replicates of long-term closed-loop control were able to maintain *P. putida* abundance at around 30% (Figure S7A), they appeared to adapt differently and required the controller to compensate in opposite directions: one had an average temperature of 33.33°C (above the critical temperature), while the other had an average temperature of 32.35°C (below the critical temperature). This exceeds the noise of the temperature sensor, which has a base accuracy of ~0.3°C. A range of phenotypic or genetic adaptations that affect measurement or actuation could be responsible: changes in the speed or dynamics of pyoverdine production, an increase in biofilm-forming ability, adaptation to a new growth temperature, adaptation to the media, adaptation to the other species, or more. In the timescales tested, the adaptation(s) have not imparted enough of a selective advantage to overwhelm the controller, which automatically compensates and maintains the composition at the desired reference.

DISCUSSION

This work demonstrated a cybernetic approach for controlling the composition of a two-species co-culture. While the interfacing of biological systems with computers to implement control has previously been described as cybergenetic,[20,39] it is often in the context of controlling the expression of genes.[40] To highlight that control of cellular behavior is achieved without genetic programming, we describe this work as cybernetic (i.e., an automatic control system with feedback). By estimating composition from measurements of OD and natural fluorescence, computing control actions, and then changing environmental conditions to favor one species or the other, the setup can track static and dynamic reference compositions. In addition to advantages highlighted by other cybernetic works such as real-time reduction in variability, independence from inoculation/mixing ratios, the ability to set arbitrary references that can be changed mid-experiment, and the ability to compensate for adaptation over time, this approach attempts to extend cybernetic methods further, removing the need for genetically engineered parts to interface between cell and computer. It also does this with commonly available bulk-culture measurements instead of relying on automated microscopy, flow cytometry, or liquid handling/sampling equipment, which might be more costly and difficult to scale (in size of reactor or number of replicates).

While other methods of actuating the composition are viable, using temperature has implications from an application perspective. In comparison to chemical methods (e.g., growth inducers, antibiotics), which might be costly at scale, cultures are generally heated to maintain a reasonable static operating temperature. The energy expended to increase the temperature above an otherwise static setpoint for composition control is partially offset whenever energy is saved to drop the temperature below the setpoint (assuming passive cooling). Particularly in growth regimes aiming to maintain a constant ratio, temperature-based actuation therefore offers the possibility of control with no changes required to the existing infrastructure/reactor, where the only additional cost is a fraction of existing heating requirements (e.g., static setpoint control in our long-term experiment was achieved with ~6% higher energy expenditure than maintaining a constant temperature with no control, Figure S7D). Despite this, temperature-based control is not suited for certain situations, e.g., in species with identical optimum temperatures. Its operation also necessitates some growth at sub-optimal temperatures and so is best suited to cultures where maximum growth rates are similar and the difference in optimum temperature is small, such that it operates in a range close to both optima. Cultures where maximum growth rates are very different and optimal temperatures are far apart may be better served by other actuation methods (e.g., regulation of growth substrates).[6,19]

Past cybernetic studies of co-culture control have often identified genetic mutation leading to loss of function as a key determinant of their longevity. In our system, a similar challenge could emerge if the critical temperature moved beyond the range of allowable temperatures or if the temperature-growth relationship for both species flattened significantly, reducing the coupling between changes in temperature and composition. This was not observed in any of our experiments, which maintained control for at least 7 days of exponential growth (>250 generations assuming an average species doubling time of 40 min), significantly longer than other cybernetically controlled bacterial co-cultures to date. Nevertheless, past studies of microbial adaptation to temperature extremes indicate that significant changes in growth optimum are possible,[41,42] though these would need to be very significant to eliminate our controller's ability to adapt. More broadly, all characteristics may drift over the course of long experiments, but combining several measurements, some of which are tied to the cellular growth rate (e.g., pyoverdine chelates iron, which is a crucial nutrient), and thus not having a single point of failure shows the broader potential of EKF-type estimation approaches to provide robustness beyond what may be possible using a single fluorescent reporter or quorum sensing molecule.

Furthermore, we first attempted to use the *P. putida* KT2440 strain but, because of its persistent biofilm formation (despite chemical and biological intervention), swapped to the OUS82 Δ*lapA* strain. While no previously reported conditions have been able to rescue the *P. putida* Δ*lapA* mutant biofilm defect,[23] in our setup, biofilms still appear after around 24 h in *P. putida* monocultures or longer in co-cultures depending on the population ratio. While the controller can compensate for the effect of biofilms, it clearly affects the system during long-term experiments, favoring *P. putida* growth until swapping into a fresh vial, at which point only planktonic cells (predominantly *E. coli*) are transferred, resulting in a sharp drop in estimated and real

composition, as observed in Figure 6C. Vial changes were done concurrently with sampling, explaining why estimated composition drops after most flow cytometry data points. However, if scaling up to larger bioreactors, the effect of biofilm on the population would be minimized—the volume of culture scales by the cube, while available surface area (and thus the relative amount of biofilm) scales by the square, making this factor less significant at scale.

Additionally, in this work, two key behaviors governing co-culture dynamics (growth rate and biofilm formation) were observed to be similar when cultivated alone or in co-culture, which facilitated our translation of monoculture models while avoiding the the complexity of modeling interspecies interactions. Here, we additionally benefited from the cybernetic control approach, which is (externally) able to mitigate several sources of variation. However, straightforward translation from monoculture to co-culture behavior cannot be assumed to be always straightforward, and, if working with microbes where such interactions are present, these should be quantified and accounted for: first, existing literature should be reviewed to identify any known interactions. Then, the growth rate should be measured in monoculture and co-culture under similar conditions to determine the presence, directionality, and magnitude of the interspecies interactions. Such pairwise experiments are commonly done to capture the effect of interspecies interactions in Lotka-Volterra modeling of microbial communities,[43–47] which has been used to predict behavior in larger microbial communities.[48,49] While a mechanistic understanding of the individual interactions (e.g., production of extracellular compounds[50]) would be ideal, the net effect of all interactions is commonly combined into a single coefficient and can be measured with straightforward growth experiments.[44] If there are no significant differences, the effect of interactions can be disregarded (as in this work), but if an effect is observed, this should also be quantified across the range of conditions used to actuate the co-culture[51] and then included in the model such that growth of one species at a given time point is expressed as a function of the abundance of the other species and the interaction coefficient at those conditions. Such models are imperfect and require additional parameterization experiments as they are applied to a varying environment. However, some of their associated weaknesses, e.g., inability to account for higher order interactions,[52] are not relevant in a two-species co-culture maintained in exponential growth phase with no nutrient limitations.

Finally, with some consideration and adjustments, this approach can be applied to other modes of bioreactor operation. For example, for continuous culturing setups such as a chemostat in steady state where dilution is matched with growth, transferability depends on whether dilution happens in large pulses (in which case it functions similarly, only requiring adjustment to the dilution term in equations) or in small pulses that are almost continuous. As this prevents the use of the time derivative of OD as an input to the EKF (which remains accurate at temperature extremes), the latter would have reduced accuracy in certain control schemes, but estimation from other data can still be sufficient, e.g., as demonstrated in Figure 5. Medium-sized pulses in a chemostat might even have improved estimation accuracy over current results as it would increase the update frequency of OD time-derivative data, though the shorter period makes it more susceptible to measurement noise. Semi-continuous fed-batch reactors require consideration on a case-by-case basis, as even if the dilution term is removed, the growth rates and measurement depend on the specific feed-profile being employed. Different experiments and adjustments to the model and estimator would therefore be needed to capture microbial behavior in such conditions.

In summary, this work presents an alternative to co-cultures that self-regulate with genetically encoded control circuitry: external cybernetic control, interfaced without genetic engineering of inter-species interactions. Each approach has strengths and weaknesses. Self-regulation is the only feasible approach for many applications outside bioreactors (e.g., nitrogen-fixing soil bacteria for crops, probiotic bacteria in the gut) but has difficulty with tracking changes in desired target composition or real-time noise rejection, such as in response to stochasticity or environmental perturbations. Cybernetic approaches that interface with engineered bacteria can overcome those weaknesses and have been applied broadly but have the associated metabolic burden and genetic instability from the engineering needed to interface cell and computer. Our cybernetic approach, while requiring additional parameterization to evaluate dynamics under different conditions, lowers this burden and the risk of control breakdown due to genetic instability. While demonstrated with *P. putida* and *E. coli*, our cybernetic approach should be generalizable to other microbes by following a similar process of identifying methods of actuating and measuring culture composition, characterizing them in monoculture, and then developing a model and controller. If using microbes with similar optimum temperatures, other characteristics could be exploited to actuate composition including differing substrate utilisation,[6,19,21] optimal nutrient concentration (e.g., halophilic *V. natriegens*[53] actuated with salt concentration), natural resistance to chemicals and antibiotics (e.g., tetracycline resistant *B. subtilis*[54]), or sensitivity to light (e.g., cyanobacteria or microalgae). Similarly, while some other microbes also produce naturally fluorescent (e.g., chlorophyll in photosynthetic microbes) or luminescent (e.g., *A. fischeri*[55]) molecules that can act as reporters for composition measurement, employing an extended Kalman filter to combine with other measurements, the curvature and absolute value of OD are strain agnostic and can always be used. Extending this approach to other species of interest should enable robust control of co-cultures for a wide range of biotechnological applications, effectively leveraging and unifying the most attractive properties of computational and biological systems.

Limitations of the study

While our approach achieved reasonable control, several areas of improvement remain for future work: specific to this co-culture, the oscillating fluorescence of *P. putida* 0US82 was investigated but not fully understood or negated, impacting estimation accuracy. Growth rates were also parameterized at a monoculture OD of 0.5, but in the co-culture, each species is at a lower OD (the sum of their ODs is 0.5), and their growth rate and dynamics might differ. On a broader level, the biofilm forming phenotype persisted despite biological and chemical

interventions and may appear when working with other species in the turbidostat environment. Additionally, because none were observed, this study did not model interspecies interactions. If present, these must be parameterized and accounted for in estimation and control. Finally, as evidenced by the effect of temperature on pyoverdine production, control that alters a cell's growth rate influences its physiological state and bioproduction capabilities. Static references where the culture is held at the critical temperature would likely be less affected, as at that temperature, *P. putida* and *E. coli* grow at 91% and 81% of their maximum growth rates, but a dynamic reference with temperatures closer to either end of the range might be more impactful. This tradeoff relies on the advantage of a robustly controlled co-culture outweighing the disadvantages of altered cell states.

RESOURCE AVAILABILITY

Lead contact

Requests for further information and resources should be directed to and will be fulfilled by the lead contact, Harrison Steel (harrison.steel@eng.ox.ac.uk).

Materials availability

CAD files for 3D printing reactor lids or media reservoir Duran bottle lids are available at Zenodo Data: https://doi.org/10.5281/zenodo.14887137.

Data and code availability

- Data from bioreactors, flow cytometers, and plate readers have been deposited at Zenodo and are publicly available at Zenodo Data: https://doi.org/10.5281/zenodo.14878543 as of the date of publication
- All original code has been deposited at Github: https://github.com/Janmorlock/CompEst3000 and Github: https://github.com/Janmorlock/ChiBio and is publicly available at Zenodo Data: https://doi.org/10.5281/zenodo.14882858 and Zenodo Data: https://doi.org/10.5281/zenodo.14882854 as of the date of publication.
- Any additional information required to reanalyze the data reported in this paper is available from the lead contact upon request.

ACKNOWLEDGMENTS

The authors thank Robert Hedley and Vasiliki Tsioligka for providing technical assistance with flow cytometry at The Don Mason Facility of Flow Cytometry at the University of Oxford. Professor Tim Tolker Nielsen and Dr. Morten Rybtke from the University of Copenhagen and Stephan Uphoff from the University of Oxford are greatly thanked for their kind gift of *P. putida* OUS82 and *E. coli* strains. This work was supported by the Engineering and Physical Sciences Research Council (grant number EP/W000326/1).

AUTHOR CONTRIBUTIONS

Conceptualization, T.A.L. and H.S.; methodology, T.A.L., J.M., and H.S.; investigation, T.A.L., J.M., and J.A.; software, J.M.; writing – original draft, T.A.L., J.M., and H.S.; writing – review & editing, T.A.L., J.M., J.A., and H.S.; funding acquisition, H.S.; supervision, H.S.

DECLARATION OF INTERESTS

The authors declare no competing interests.

STAR★METHODS

Detailed methods are provided in the online version of this paper and include the following:

- KEY RESOURCES TABLE
- EXPERIMENTAL MODEL AND STUDY PARTICIPANT DETAILS
  - Microbial strains
  - Growth and storage
- METHOD DETAILS
  - Fluorescence scan
  - Bioreactor setup
  - Experimental setup
  - Offline validation
  - Biofilm assay
  - Models in state estimation
- QUANTIFICATION AND STATISTICAL ANALYSIS

SUPPLEMENTAL INFORMATION

Supplemental information can be found online at https://doi.org/10.1016/j.crmeth.2025.101009.

Received: August 7, 2024
Revised: January 20, 2025
Accepted: February 25, 2025
Published: March 24, 2025

REFERENCES

1. Shin, J., Liao, S., Kuanyshev, N., Xin, Y., Kim, C., Lu, T., and Jin, Y.S. (2024). Compositional and temporal division of labor modulates mixed sugar fermentation by an engineered yeast consortium. Nat. Commun. *15*, 781. https://doi.org/10.1038/s41467-024-45011-w.
2. Tsoi, R., Wu, F., Zhang, C., Bewick, S., Karig, D., and You, L. (2018). Metabolic division of labor in microbial systems. Proc. Natl. Acad. Sci. USA *115*, 2526–2531. https://doi.org/10.1073/pnas.1716888115.
3. Gasparek, M., Steel, H., and Papachristodoulou, A. (2023). Deciphering mechanisms of production of natural compounds using inducer-producer microbial consortia. Biotechnol. Adv. *64*, 108117. https://doi.org/10.1016/j.biotechadv.2023.108117.
4. Stenuit, B., and Agathos, S.N. (2015). Deciphering microbial community robustness through synthetic ecology and molecular systems synecology. Curr. Opin. Biotechnol. *33*, 305–317. https://doi.org/10.1016/j.copbio.2015.03.012.
5. Xia, T., Eiteman, M.A., and Altman, E. (2012). Simultaneous utilization of glucose, xylose and arabinose in the presence of acetate by a consortium of Escherichia coli strains. Microb. Cell Fact. *11*, 77. https://doi.org/10.1186/1475-2859-11-77.
6. Martinez, J.A., Delvenne, M., Henrion, L., Moreno, F., Telek, S., Dusny, C., and Delvigne, F. (2022). Controlling microbial co-culture based on substrate pulsing can lead to stability through differential fitness advantages. PLoS Comput. Biol. *18*, e1010674. https://doi.org/10.1371/journal.pcbi.1010674.
7. Hardin, G. (1960). The Competitive Exclusion Principle. Science *131*, 1292–1297. https://doi.org/10.1126/science.131.3409.1292.
8. Dinh, C.V., Chen, X., and Prather, K.L.J. (2020). Development of a Quorum-Sensing Based Circuit for Control of Coculture Population Composition in a Naringenin Production System. ACS Synth. Biol. *9*, 590–597. https://doi.org/10.1021/acssynbio.9b00451.
9. Salma, A., Abdallah, R., Fourcade, F., Amrane, A., and Djelal, H. (2021). A New Approach to Produce Succinic Acid Through a Co-Culture System. Appl. Biochem. Biotechnol. *193*, 2872–2892. https://doi.org/10.1007/s12010-021-03572-2.
10. Mee, M.T., Collins, J.J., Church, G.M., and Wang, H.H. (2014). Syntrophic exchange in synthetic microbial communities. Proc. Natl. Acad. Sci. USA *111*, E2149–E2156. https://doi.org/10.1073/pnas.1405641111.

CellPress
OPEN ACCESS

11. Miano, A., Liao, M.J., and Hasty, J. (2020). Inducible cell-to-cell signaling for tunable dynamics in microbial communities. Nat. Commun. *11*, 1193. https://doi.org/10.1038/s41467-020-15056-8.

12. Boo, A., Mehta, H., Amaro, R.L., and Stan, G.B. (2023). Host-aware RNA-based control of synthetic microbial consortia. Preprint at bioRxiv. https://doi.org/10.1101/2023.05.15.540816.

13. McCarty, N.S., and Ledesma-Amaro, R. (2019). Synthetic Biology Tools to Engineer Microbial Communities for Biotechnology. Trends Biotechnol. *37*, 181–197. https://doi.org/10.1016/J.TIBTECH.2018.11.002.

14. Caringella, G., Bandiera, L., and Menolascina, F. (2023). Recent advances, opportunities and challenges in cybergenetic identification and control of biomolecular networks. Curr. Opin. Biotechnol. *80*, 102893. https://doi.org/10.1016/j.copbio.2023.102893.

15. Treloar, N.J., Fedorec, A.J.H., Ingalls, B., and Barnes, C.P. (2020). Deep reinforcement learning for the control of microbial co-cultures in bioreactors. PLoS Comput. Biol. *16*, e1007783. https://doi.org/10.1371/journal.pcbi.1007783.

16. Bertaux, F., Sosa-Carrillo, S., Gross, V., Fraisse, A., Aditya, C., Furstenheim, M., and Batt, G. (2022). Enhancing bioreactor arrays for automated measurements and reactive control with ReacSight. Nat. Commun. *13*, 3363, Group. https://doi.org/10.1038/s41467-022-31033-9.

17. Aditya, C., Bertaux, F., Batt, G., and Ruess, J. (2021). A light tunable differentiation system for the creation and control of consortia in yeast. Nat. Commun. *12*, 5829. https://doi.org/10.1038/s41467-021-26129-7.

18. Gutiérrez Mena, J., Kumar, S., and Khammash, M. (2022). Dynamic cybergenetic control of bacterial co-culture composition via optogenetic feedback. Nat. Commun. *13*, 4808. https://doi.org/10.1038/s41467-022-32392-z.

19. Martinez, J.A., Bouchat, R., Aurin, T.G.d.S., Martínez, L.M., Caspeta, L., Telek, S., Zicler, A., Gosset, G., and Delvigne, F. (2024). Automated adjustment of metabolic niches enables the control of natural and engineered microbial co-cultures. Preprint at bioRxiv. https://doi.org/10.1101/2024.05.14.594082.

20. Lee, T.A., and Steel, H. (2022). Cybergenetic control of microbial community composition. Front. Bioeng. Biotechnol. *10*, 957140. https://doi.org/10.3389/fbioe.2022.957140.

21. Kusuda, M., Shimizu, H., and Toya, Y. (2021). Reactor control system in bacterial co-culture based on fluorescent proteins using an Arduino-based home-made device. Biotechnol. J. *16*, 2100169. https://doi.org/10.1002/biot.202100169.

22. Steel, H., Habgood, R., Kelly, C.L., and Papachristodoulou, A. (2020). In situ characterisation and manipulation of biological systems with Chi.Bio. PLoS Biol. *18*, e3000794. https://doi.org/10.1371/JOURNAL.PBIO.3000794.

23. Ainelo, H., Lahesaare, A., Teppo, A., Kivisaar, M., and Teras, R. (2017). The promoter region of lapA and its transcriptional regulation by Fis in Pseudomonas putida. PLoS One *12*, e0185482. https://doi.org/10.1371/journal.pone.0185482.

24. Gjermansen, M., Nilsson, M., Yang, L., and Tolker-Nielsen, T. (2010). Characterization of starvation-induced dispersion in Pseudomonas putida biofilms: genetic elements and molecular mechanisms. Mol. Microbiol. *75*, 815–826. https://doi.org/10.1111/j.1365-2958.2009.06793.x.

25. Hoegy, F., Mislin, G.L.A., and Schalk, I.J. (2014). Pyoverdine and Pyochelin Measurements. In Pseudomonas Methods and Protocols, A. Filloux and J.L. Ramos, eds. (Springer), pp. 293–301. https://doi.org/10.1007/978-1-4939-0473-0_24.

26. Krieger, A.G., Zhang, J., and Lin, X.N. (2021). Temperature regulation as a tool to program synthetic microbial community composition. Biotechnol. Bioeng. *118*, 1381–1392. https://doi.org/10.1002/bit.27662.

27. Kaplan, J.B. (2010). Biofilm Dispersal. J. Dent. Res. *89*, 205–218. https://doi.org/10.1177/0022034509359403.

28. Van den Bergh, B., Swings, T., Fauvart, M., and Michiels, J. (2018). Experimental Design, Population Dynamics, and Diversity in Microbial Experimental Evolution. Microbiol. Mol. Biol. Rev. *82*, e00008-18. https://doi.org/10.1128/mmbr.00008–18.

29. Gramelsberger, G. (2018). Continuous culture techniques as simulators for standard cells: Jacques Monod's, Aron Novick's and Leo Szilard's quantitative approach to microbiology. Hist. Philos. Life Sci. *40*, 23. https://doi.org/10.1007/s40656-017-0182-x.

30. Espeso, D.R., Dvořák, P., Aparicio, T., and de Lorenzo, V. (2020). An automated DIY framework for experimental evolution of Pseudomonas putida. Microb. Biotechnol. *14*, 2679–2685. https://doi.org/10.1111/1751-7915.13678.

31. Yildiz, M.S. (2021). Understanding the Limits on Exponential Growth of Bacteria through Long Term Evolution. PhD Thesis (Harvard University). https://www.proquest.com/docview/2564494350/abstract/5C002F239DE64AFEPQ/1.

32. Sloup, R.E., Cieza, R.J., Needle, D.B., Abramovitch, R.B., Torres, A.G., and Waters, C.M. (2016). Polysorbates prevent biofilm formation and pathogenesis of Escherichia coli O104:H4. Biofouling *32*, 1131–1140. https://doi.org/10.1080/08927014.2016.1230849.

33. Kamali, E., Jamali, A., Izanloo, A., and Ardebili, A. (2021). In vitro activities of cellulase and ceftazidime, alone and in combination against Pseudomonas aeruginosa biofilms. BMC Microbiol. *21*, 347. https://doi.org/10.1186/s12866-021-02411-y.

34. Barrientos-Moreno, L., Molina-Henares, M.A., Pastor-García, M., Ramos-González, M.I., and Espinosa-Urgel, M. (2019). Arginine Biosynthesis Modulates Pyoverdine Production and Release in Pseudomonas putida as Part of the Mechanism of Adaptation to Oxidative Stress. J. Bacteriol. *201*, e00454-19. https://doi.org/10.1128/JB.00454-19.

35. Fleurial, M., Sacchelli, L., and Yabo, A.G. (2024). State estimation in alcoholic fermentation models: a case-study in wine-making conditions. . 2024 European Control Conference (ECC) https://doi.org/10.23919/ECC64448.2024.10591226.

36. Hellmann, S., Wilms, T., Streif, S., and Weinrich, S. (2024). Comparison of Unscented Kalman Filter Design for Agricultural Anaerobic Digestion Model. Preprint at arXiv. https://doi.org/10.48550/arXiv.2310.15958.

37. Asswad, R., Cinquemani, E., and Gouzé, J.L. (2024). Kalman-based approaches for online estimation of bioreactor dynamics from fluorescent reporter measurements. Preprint at arXiv. https://doi.org/10.48550/arXiv.2404.16649.

38. Åström, K.J., and Murray, R. (2021). Feedback Systems: An Introduction for Scientists and Engineers, Second Edition (Princeton University Press).

39. Khammash, M., Di Bernardo, M., and Di Bernardo, D. (2019). Cybergenetics: Theory and Methods for Genetic Control System. In 2019 IEEE 58th Conference on Decision and Control (CDC), pp. 916–926. https://doi.org/10.1109/CDC40024.2019.9030209.

40. Carrasco-López, C., García-Echauri, S.A., Kichuk, T., and Avalos, J.L. (2020). Optogenetics and biosensors set the stage for metabolic cybergenetics. Curr. Opin. Biotechnol. *65*, 296–309. https://doi.org/10.1016/j.copbio.2020.07.012.

41. Toll-Riera, M., Olombrada, M., Castro-Giner, F., and Wagner, A. (2022). A limit on the evolutionary rescue of an Antarctic bacterium from rising temperatures. Sci. Adv. *8*, eabk3511. https://doi.org/10.1126/sciadv.abk3511.

42. Lehmann, M., Prohaska, C., Zeldes, B., Poehlein, A., Daniel, R., and Basen, M. (2023). Adaptive laboratory evolution of a thermophile toward a reduced growth temperature optimum. Front. Microbiol. *14*, 1265216. https://doi.org/10.3389/fmicb.2023.1265216.

43. Dorado-Morales, P., Vilanova, C., P. Garay, C., Martí, J.M., and Porcar, M. (2015). Unveiling Bacterial Interactions through Multidimensional Scaling and Dynamics Modeling. Sci. Rep. *5*, 18396. https://doi.org/10.1038/srep18396.

44. Davis, J.D., Olivença, D.V., Brown, S.P., and Voit, E.O. (2022). Methods of quantifying interactions among populations using Lotka-Volterra models.

Frontiers in Systems Biology *2*, 1021897. https://doi.org/10.3389/fsysb.2022.1021897.

45. Fisher, C.K., and Mehta, P. (2014). Identifying Keystone Species in the Human Gut Microbiome from Metagenomic Timeseries Using Sparse Linear Regression. PLoS One *9*, e102451. https://doi.org/10.1371/journal.pone.0102451.
46. Freilich, S., Zarecki, R., Eilam, O., Segal, E.S., Henry, C.S., Kupiec, M., Gophna, U., Sharan, R., and Ruppin, E. (2011). Competitive and cooperative metabolic interactions in bacterial communities. Nat. Commun. *2*, 589. https://doi.org/10.1038/ncomms1597.
47. Joseph, T.A., Shenhav, L., Xavier, J.B., Halperin, E., and Pe'er, I. (2020). Compositional Lotka-Volterra describes microbial dynamics in the simplex. PLoS Comput. Biol. *16*, e1007917. https://doi.org/10.1371/journal.pcbi.1007917.
48. Guo, X., and Boedicker, J.Q. (2016). The Contribution of High-Order Metabolic Interactions to the Global Activity of a Four-Species Microbial Community. PLoS Comput. Biol. *12*, e1005079. https://doi.org/10.1371/journal.pcbi.1005079.
49. Stein, R.R., Bucci, V., Toussaint, N.C., Buffie, C.G., Rätsch, G., Pamer, E.G., Sander, C., and Xavier, J.B. (2013). Ecological Modeling from Time-Series Inference: Insight into Dynamics and Stability of Intestinal Microbiota. PLoS Comput. Biol. *9*, e1003388. https://doi.org/10.1371/journal.pcbi.1003388.
50. Hibbing, M.E., Fuqua, C., Parsek, M.R., and Peterson, S.B. (2010). Bacterial competition: surviving and thriving in the microbial jungle. Nat. Rev. Microbiol. *8*, 15–25. https://doi.org/10.1038/nrmicro2259.
51. Song, H.S., Lee, N.R., Kessell, A.K., McCullough, H.C., Park, S.Y., Zhou, K., and Lee, D.Y. (2024). Kinetics-based inference of environment-dependent microbial interactions and their dynamic variation. mSystems *9*, e0130523. https://doi.org/10.1128/msystems.01305-23.
52. Ishizawa, H., Tashiro, Y., Inoue, D., Ike, M., and Futamata, H. (2024). Learning beyond-pairwise interactions enables the bottom–up prediction of microbial community structure. Proc. Natl. Acad. Sci. USA *121*, e2312396121. https://doi.org/10.1073/pnas.2312396121.
53. Weinstock, M.T., Hesek, E.D., Wilson, C.M., and Gibson, D.G. (2016). Vibrio natriegens as a fast-growing host for molecular biology. Nat. Methods *13*, 849–851. https://doi.org/10.1038/nmeth.3970.
54. Bechhofer, D.H., and Stasinopoulos, S.J. (1998). tetA(L) Mutants of a Tetracycline-Sensitive Strain of Bacillus subtilis with the Polynucleotide Phosphorylase Gene Deleted. J. Bacteriol. *180*, 3470–3473. https://www.ncbi.nlm.nih.gov/pmc/articles/PMC107306/.
55. Miyashiro, T., and Ruby, E.G. (2012). Shedding light on bioluminescence regulation in Vibrio fischeri. Mol. Microbiol. *84*, 795–806. https://doi.org/10.1111/j.1365-2958.2012.08065.x.

STAR★METHODS

KEY RESOURCES TABLE

REAGENT or RESOURCE	SOURCE	IDENTIFIER
Bacterial and virus strains		
P. putida KT2440	American Type Culture Collection	Cat#47054
P. putida OUS82 -lapA	Provided by the group of Prof.Tim Tolker-Nielsen	OUS82 ΔlapA
E. coli MG1655	American Type Culture Collection	Cat#47076
E. coli MG1655-RFP	Provided by the group of Prof. Stephan Uphoff	MG1655 pRNA1-mKate2
Chemicals, peptides, and recombinant proteins		
M9 Minimal Salts	Formedium	Cat#MMS0102
Glucose	Formedium	Cat#GLU03
$MgSO_4$	Sigma Aldrich	Cat#M7506-500G
$CaCl_2$	Sigma Aldrich	Cat#C5670-500G
EDTA-Na_2	Sigma Aldrich	Cat#E4884-100G
$FeCl_3$	Sigma Aldrich	Cat#157740-100G
$ZnCl_2$	Sigma Aldrich	Cat#208086-100G
$CuCl_2.2H_2O$	Sigma Aldrich	Cat#307483-500G
$CoCl_2$	Sigma Aldrich	Cat#232696-100G
H_3BO_3	Sigma Aldrich	Cat#B0394-500G
$MnCl_2 \cdot 4H_2O$	Sigma Aldrich	Cat#221279-500G
Casamino Acids	Formedium	Cat#CAS03
Arginine	Formedium	Cat#DOC0108
Serine	Formedium	Cat#DOC0180
Glutamine	Formedium	Cat#DOC0132
CSM Double Drop-Out -Arg, -Trp	Formedium	Cat#DCS0449
Glycerol	Sigma Aldrich	Cat#G5516-1L
PBS	Sigma Aldrich	Cat#P3813-1PAK
Software and algorithms		
Chi.Bio custom OS	This study	Zenodo: https://doi.org/10.5281/zenodo.14882854
Composition estimator	This study	Zenodo: https://doi.org/10.5281/zenodo.14882858
Other		
Aria III flow cytometer	BD Biosciences	BD FACSAria™ III
Chi.Bio bioreactor	Labmaker	Chi.Bio Reactor Only (7-color LED)
Chi.Bio pump board and control computer	Labmaker	Chi.Bio (5-color LED)
3D printed reactor lid	This study	Zenodo: https://doi.org/10.5281/zenodo.14887137
3D printed Duran bottle lid	This study	Zenodo: https://doi.org/10.5281/zenodo.14887137
Reactor vial	Fisher Scientific	Cat#11593532
Media tubes	Altec	Cat#01-93-1416/20
Stir bar	Fisher Scientific	Cat#11818862
1-way valve	Mc-Master Carr	Cat#7757K41
0.2 μm filter	Fisher Scientific	Cat#15206869

EXPERIMENTAL MODEL AND STUDY PARTICIPANT DETAILS

Microbial strains

Unless specified, the strains are *P. putida* OUS82 with a *lapA* gene knockout and *E. coli* MG1655 with a genomically encoded mKate2 cassette with a PRNA1 promoter inserted at a neutral TN7 site. The 2D fluorescent scan was done with wild type *P. putida* KT2440 and wild type *E. coli* MG1655. Wild type *P. putida* KT2440 strain was also used in some experiments shown in the "Challenges in co-culture implementation" section.

Growth and storage

Strains are stored in 25% glycerol at -80°C and revived by streaking overnight onto LB-agar incubated at 30°C (for *P. putida*) or 37°C (for *E. coli*). Unless specified, all growth was done in M9 (Formedium) supplemented with 0.4% glucose (Formedium), 2 mM Mg_2SO_4 (Sigma Aldrich), 0.1 mM $CaCl_2$ (Sigma Aldrich), and trace elements (final concentrations of 50 mg L^{-1} EDTA.NA_2.2H_2O, 30.8 μM $FeCl_3$, 6.16 μM $ZnCl_2$, 0.76 μM $CuCl_2$.2H_2O, 0.42 μM $CoCl_2$, 1.62 μM H_3BO_3, and 0.081 μM $MnCl_2$.4H_2O, all from Sigma Aldrich). For some experiments in the "Challenges in co-culture implementation" the media was supplemented with a range of CAA from Formedium, but unless specified all other experiments used a final concentration of 0.02% CAA. For experiments exploring the effect of CAA or other amino acids on oscillations, arginine, serine, glutamine, and Complete Synthetic Mixture (CSM) with double dropouts for arginine and tryptophan (all from Formedium) were used. When spiking with amino acids, they are added to the same specified concentration in the reactor and media bottle.

METHOD DETAILS

Fluorescence scan

1 mL of *P. putida* and *E. coli* were grown overnight at 30/37°C shaking at 200 RPM, washed and resuspended in PBS (Formedium), and then 200 μL pipetted into the wells of a 96 well plate (Greiner Bio-One). This was measured in a Tecan Spark plate reader in "Fluorescence Intensity 3D Scan Top Reading" mode with an excitation and emission wavelength range of 280–900 nm with a step size of 20 nm, bandwidth of 20 nm, manual gain of 100, and 30 flashes each.

Bioreactor setup

To benchmark 440 nm fluorescence measurements between reactors, each bioreactor was associated with a unique identifier and then calibrated with a dilution range of a filter-sterilised *P. putida* monoculture grown to stationary phase (removing cells which might scatter/absorb light, but retaining extracellular pyoverdine). The un-normalised fluorescence values were collected after stirring for 5 s and settling for 5 s, mimicking experimental conditions. For control experiments, the identifiers of the reactors being used were entered into the control computer, and the controller used the unique reactor offsets and scaling factors to calculate a normalised fluorescence value. The Chi.Bio OS and custom program with these functionalities and the real-time composition estimator is available online (see Data Availability). For OD readings, before each experiment, all vials were autoclaved and wiped with ethanol, filled with media, heated to 33.2°C with stirring on, and then used as a blank.

To calculate growth rates for parameterisation or during control experiments, the OD of the culture was "dithered" around a setpoint of 0.5, where it was allowed to grow to 0.545 before being diluted to 0.455. The growth rate was calculated from the logarithmic slope between these two points. These values were selected instead of the default Chi.Bio dithering parameters to increase the frequency of dilutions, which improved the performance of the estimator. Parameterisation was done by growing for 12 h at one temperature before swapping to another, and calculating the average growth rate after the culture fully adjusts to the new temperature. All bioreactor parameters that are not specified (e.g., stir speed) are left at default values.

3D-printed lids (see data availability) were used to cap reactors with the culture and duran bottles containing fresh media. To prevent negative pressure from pumping out fresh media during the experiment, an ethanol sterilised tube with a 0.22 μm filter (Fisher Scientific) was connected to the lid to allow sterile air into the fresh media reservoirs. The bioreactor vial lids contain a similar tube and filter for proper aeration of the cultures, as fresh air is constantly sucked in to the culture head-space as the waste pump pulls air out. In longer experiments, a one-way valve (McMaster-Carr) was installed on the media inlet tube, as any backflow would allow aerosolised bacteria to enter the tube. The valves and tubes were sterilised daily with ethanol.

Experimental setup

All bioreactor experiments are started by plating a glycerol stock onto LB-agar and growing overnight at 30/37°C for *P. putida*/*E. coli* respectively. A single colony is used to inoculate a 5 mL pre-culture with the same media as the experiment (to reduce effects of metabolism adapting to the media) and incubated shaking at 30/37°C to mid-exponential phase for 4–8 h (depending on size and age of the colony used). For inoculation, the pre-culture OD is measured, 1 mL of each is centrifuged with an relative centrifugal force (RCF) of 4000 for 4 min, and they are resuspended in PBS to reach an OD of 2 to remove pre-culture media and standardise the inoculation concentration. Each bioreactor, which has a culture volume of 20 mL, is inoculated with 100 μL of this PBS suspension for a final OD of 0.01.

All co-culture experiments were conducted by mixing a pair of *P. putida* and *E. coli* monocultures at steady state in a 50:50 ratio unless stated otherwise. The OD of each culture was first measured with an external spectrometer to ensure they were similar, and they were diluted with M9 otherwise. For increased standardisation, a pair of monocultures was typically mixed into 4 bioreactors: from a 20 mL culture, 16 mL was split into 4 freshly autoclaved/wiped vials, and 1 mL used for measuring OD. Each mixture, which contains a total of 8 mL, is then filled to 20 mL with media. The monocultures were typically grown at an OD of 0.75 (such that the OD after splitting into 4 reactors was around 0.375) without dithering, as they would have different ODs depending on how recently they had been diluted. Before implementing control, the culture is allowed to grow for 2 h at 33.2°C (the critical temperature) to allow it to reach the OD setpoint and stabilise. While arbitrary references are possible, for long-term experiments we selected a 30:70 *P putida*:*E. coli* ratio in an attempt to reduce the impact of biofilm formation.

Offline validation

Sampling for offline validation was done by pausing the bioreactors and removing 1 mL of culture in a sterile hood. This was centrifuged with an (RCF) of 14000 for 2 min, resuspended in 0.5 mL of PBS, then mixed with 0.5 mL of 50% glycerol and stored at -80°C. For validation, samples were thawed on 4°C metal beads, then washed once and resuspended in 1 mL of 4°C PBS. For flow cytometry, samples were analyzed in a FACSAriaIII (BD Biosciences) with a 405 nm laser and 450/40 nm filter for pyoverdine and a 561 nm laser and 610/20 nm filter for RFP. Voltages were as follows: 400 (FSC), 350 (SSC), 650 (561–610/20), and 400 (405-405/40). FSC-A and SSC-A channels were used to gate living cells, and for validating control experiments *P. putida* or *E. coli* monocultures were used as negative/positive controls for fluorescence with the 561 nm laser. 10,000 events were collected per measurement, of which around half were typically gated as living cells. This was lower than those of fresh samples and was attributed to freeze-thaw from sample storage. More events could be collected to overcome the negative effects of storage. Flow cytometry noise was calculated from measurements of 12 replicates of samples taken immediately after mixing (Figure S3B). For plating, samples were diluted serially 8 times in a 96 well plate with PBS. 5 μL of each dilution was then plated onto two different LB-agar plates, one containing 15 μg mL^{-1} chloramphenicol. Colonies from the least diluted level were counted, where *E. coli* = colonies without Cm - colonies with Cm.

Biofilm assay

Biofilms were stained with 0.1% w/v crystal violet for visualisation and assays. Vials or 96 well plates containing biofilms are emptied of culture by shaking over a waste tray, washed by gently submerging in tap water, then left to dry in a sterile hood for 15 min before staining for 10 min (22 mL for vials and 220 μL for plates). The stain was poured into a waste tray, and the biofilms were washed thrice by gently submerging them in tap water before being left to dry and stored. For quantification, 95% ethanol was used to solubilise the crystal violet from the biofilms for 15 min, then 125 μL was transferred to a 96 well plate and OD_{595} measured in a platereader. To evaluate the effect of Tween (polysorbate) 20 or cellulase on biofilm formation (Figure S4), they were added in a range of concentrations to 20 mL of culture in bioreactors or to 200 μL of culture in 96 well plates and grown overnight (bioreactor) or for 24 h (plates).

Models in state estimation

System model

The system model governs the dynamics of three key components, i.e., the $E(t)$ represents the OD of *E. coli*, $P(t)$ the OD of *P. putida*, and $F[k]$ the fluorescence level of pyoverdine. To better observe bacteria growth and pyoverdine production, and hence derive a more accurate composition estimate, the OD was dithered around a setpoint. This resulted in a growth period without any dilution and rising OD, and a dilution period where OD is reduced until a lower threshold is reached. As such, the system model was split into growth and dilution models to account for the different phases of the reactor and thus co-culture dynamics.

For growth, it was assumed that substrates in the media are present in excess of their limiting concentration and changes in their concentration are thus negligible. At low OD, bacterial growth can be modeled by:

$$\begin{aligned} \dot{P}(t) &= \mu_P(t)P(t) \\ \dot{E}(t) &= \mu_E(t)E(t) \end{aligned} \qquad \text{(Equation 1)}$$

where the temperature-dependent and hence time-dependent growth rate $\mu(t)$ describes how fast the OD of the individual culture increase at time t. Similar to the state variables $E(t)$ and $P(t)$, the growth rates relate to the change of bacteria OD over time and do not describe the cell's individual growth. Euler Forward discretization of the ordinary differential equations (ODEs) in Equation 1 with sampling time t_s results in:

$$\begin{aligned} P[k+1] &= (1 + t_s(\mu_P[k] + \nu_P[k]))P[k] \\ E[k+1] &= (1 + t_s(\mu_E[k] + \nu_E[k]))E[k] \end{aligned} \qquad \text{(Equation 2)}$$

with process noise $\nu_P[k]$ and $\nu_E[k]$ at cycle k. Assuming that the production of pyoverdine only linearly depends on the present amount of *P. putida* and production rate $\mu_F[k]$, pyoverdine fluorescence $F[k]$ with process noise $\nu_F[k]$ can be modeled as follows:

$$F[k+1] = F[k] + (t_s(\mu_F[k] + \nu_F[k]))P[k] \qquad \text{(Equation 3)}$$

The process noise is multiplicative to model the increased absolute uncertainty at higher bacteria abundance. Intuitively, it describes the uncertainty of the defined growth and production rates.

Temperature's influence on growth and production rates were determined in separate monocultures through OD and fluorescence measurements between dilutions. The detailed computation of the rates from the measurements is described in depth in later sections. The measured rates converge to a steady state for each temperature after passing a transient phase. By fitting polynomial models, we obtained the steady-state bacterial growth rates $\mu_{P,ss}[T]$, $\mu_{E,ss}[T]$, and $\mu_{F,ss}[T]$ as a function of the temperature T. To accurately describe the transient phase of the *P. putida* and *E. coli* growth rates we add dynamics to the growth and production rates with a first-order infinite impulse response (IIR) filter:

$$\begin{aligned} \mu_P[k+1] &= \mu_P[k] + \alpha_P\left(\mu_{P,ss}[T] - \mu_P[k]\right) \\ \mu_E[k+1] &= \mu_E[k] + \alpha_E\left(\mu_{E,ss}[T] - \mu_E[k]\right) \end{aligned} \qquad \text{(Equation 4)}$$

The pyoverdine production rate displayed damped oscillations after a temperature change. These were modeled by extending the IIR filter by a lag of k_d cycles:

$$\mu_F[k+1] = \mu_F[k] + \alpha_F\left(\mu_{F,ss}[T] - \mu_F[k-k_d]\right) \quad \text{(Equation 5)}$$

Due to the observed variation of these oscillations in magnitude and period, a conservative model that did not match the observed data perfectly was chosen to limit the risk of overfitting.

For dilution, in the chosen reactor configuration, OD dropped on average by r_{dil} per cycle during dilution. Hence, each quantity decreased as described in the dilution model with multiplicative process noise $\nu_{P,dil}$, $\nu_{E,dil}$, and $\nu_{F,dil}$:

$$\begin{aligned} P[k+1] &= P[k]\left(1 - \frac{r_{dil}}{E[k]+P[k]} + \nu_{P,dil}\right) \\ E[k+1] &= E[k]\left(1 - \frac{r_{dil}}{E[k]+P[k]} + \nu_{E,dil}\right) \\ F[k+1] &= F[k]\left(1 - \frac{r_{dil}}{E[k]+P[k]} + \nu_{F,dil}\right) \end{aligned} \quad \text{(Equation 6)}$$

In the dilution period, these equations are added to the growth period model. All introduced process noises

$$\boldsymbol{\nu} = \begin{pmatrix} \nu_P \\ \nu_E \\ \nu_F \end{pmatrix} \quad \boldsymbol{\nu}_{dil} = \begin{pmatrix} \nu_{P,dil} \\ \nu_{E,dil} \\ \nu_{F,dil} \end{pmatrix}$$

are modeled to be zero-mean and mutually independent, i.e.,:

$$\mathbb{E}(\boldsymbol{\nu}) = 0 \qquad \mathbb{E}(\boldsymbol{\nu}_{dil}) = 0$$

$$\mathrm{Cov}(\boldsymbol{\nu}) = \mathbf{Q} = \begin{pmatrix} \sigma_P^2 & 0 & 0 \\ 0 & \sigma_E^2 & 0 \\ 0 & 0 & \sigma_F^2 \end{pmatrix} \quad \mathrm{Cov}(\boldsymbol{\nu}_{dil}) = \mathbf{Q}_{dil} = \begin{pmatrix} \sigma_{P,dil}^2 & 0 & 0 \\ 0 & \sigma_{E,dil}^2 & 0 \\ 0 & 0 & \sigma_{F,dil}^2 \end{pmatrix}$$

Measurement model

The measurement models are also needed for composition estimation and simulation. The Chi.Bio measures culture temperature, OD, and fluorescence once per cycle. OD is treated as a straightforward measurement assumed to be directly proportional to the combined bacteria OD. After sensor calibration, it is modeled with measurement noise ω_{od} as follows:

$$od[k] = P[k] + E[k] + \omega_{od} \quad \text{(Equation 7)}$$

Bulk fluorescence measurements, $fl[k]$, are acquired by exciting the consortia at 395 nm with light intensity I_{ex} and measuring fluorescence at an emission band around 440 nm. Broad LED emission spectra, imperfect light filters, and light scattering cause the measurement to be affected by bacterial abundance, i.e., OD. This relationship is assumed to be linear, with proportionality constant c_{od}. This together with measurement noise ω_{fl} results in:

$$fl[k] = F[k] + c_{od}od[k] + \omega_{fl} \quad \text{(Equation 8)}$$

The parameter c_{od} was obtained from fluorescence measurements under changing OD and in the absence of pyoverdine ($F[k] = 0$). The introduced measurement noise

$$\boldsymbol{\omega} = \begin{pmatrix} \omega_{od} \\ \omega_{fl} \end{pmatrix}$$

was modeled to be zero-mean and mutually independent, i.e.,:

$$\mathbb{E}(\boldsymbol{\omega}) = 0 \quad \mathrm{Cov}(\boldsymbol{\omega}) = \begin{pmatrix} \sigma_{od}^2 & 0 \\ 0 & \sigma_{fl}^2 \end{pmatrix} \quad \text{(Equation 9)}$$

Although most noise is presumably caused by inhomogeneities in the culture liquid (e.g., through precipitation), mutual independence was assumed as OD and fluorescence is measured by different sensors and at different time instances.

State estimation

An EKF was employed that computed the state estimate with unimodal distribution. Linearization around the current estimate did not guarantee optimality, however, smooth system dynamics promised near optimality with the available models and measurements. The EKF computes the estimate in two steps: the prediction step and the measurement update step.

For the prediction step, in every cycle (i.e., every minute), the EKF first predicts the current state based on the estimate from the last cycle and the production models derived in Equations 2 and 3. To minimize discretization errors, $t_s = 1$ s is used. Consequently, the

prediction step runs 60 times each cycle. The dilution models in Equation 6 were added to the prediction when the bioreactor was diluting media to maintain the desired OD (typically set to 0.5).

For the measurement update step, i.e., every last cycle of the growth period k_h, the measurement update step was augmented by intermediate estimates ($\widehat{P}_{od}[k_h]$, $\widehat{E}_{od}[k_h]$, and $\widehat{P}_{fl}[k_h]$) derived from all the measurements in the prior growth period.

The estimates through OD curvature (i.e., total growth rate) $\widehat{P}_{od}[k_h]$ and $\widehat{E}_{od}[k_h]$ are obtained by inserting the solution of Equation 1 into Equation 7:

$$\begin{aligned} od[k] &= P[k_h]e^{\mu_P(t[k]-t[k_h])} + E[k_h]e^{\mu_E(t[k]-t[k_h])} \\ &= P[k_h]e^{\mu_P(t[k]-t[k_h])} + (od[k_h] - P[k_h])e^{\mu_E(t[k]-t[k_h])} \end{aligned} \qquad \text{(Equation 10)}$$

Rearranging Equation 10 and gathering each measurement from the first cycle k_l to the last cycle k_h of the growth period into vectors results in:

$$\begin{pmatrix} e^{\mu_P(t[k_l]-t[k_h])} - e^{(\mu_E(t[k_l]-t[k_h])} \\ \vdots \\ e^{\mu_P(t[k_h]-t[k_h])} - e^{(\mu_E(t[k_h]-t[k_h])} \end{pmatrix} P[k_h] = \begin{pmatrix} od[k_l] - od[k_h]e^{\mu_E(t[k_l]-t[k_h])} \\ \vdots \\ od[k_h] - od[k_h]e^{\mu_E(t[k_h]-t[k_h])} \end{pmatrix} \qquad \text{(Equation 11)}$$

In the matrix representation $\mathbf{A}P[k_h] = \mathbf{b}$, the least squares method can be applied to obtain $\widehat{P}_{od}[k_h]$ that minimizes the residual in Equation 11. Finally, the algebraic relation in Equation 7 provides $\widehat{P}_{od}[k_h]$.

The *P. putida* estimate $\widehat{P}_{fl}[k_h]$ obtained through the curvature of the measured fluorescence is derived from the continuous model of Equation 3 and the solution of Equation 1:

$$\dot{F}(t) = \mu_F(t)P(t) = \mu_F P_h e^{\mu_P(t-t_h)} \qquad \text{(Equation 12)}$$

Integration from all cycles within the growth period to cycle k_h and rearrangement yields:

$$\begin{pmatrix} \frac{\mu_F}{\mu_P}\left(e^{\mu_P(t[k_l]-t[k_h])} - 1\right) \\ \vdots \\ \frac{\mu_F}{\mu_P}\left(e^{\mu_P(t[k_h]-t[k_h])} - 1\right) \end{pmatrix} P[k_h] = \begin{pmatrix} F[k_l] - F[k_h] \\ \vdots \\ F[k_h] - F[k_h] \end{pmatrix} \qquad \text{(Equation 13)}$$

Applying least-squares minimisation gives another *P. putida* estimate $\widehat{P}_{fl}[k_h]$.

Having a good approximation of each method's exactness is vital when combining them. If the individual measurement updates are conflicting, the EKF should prioritize the more accurate one to derive a correct composition estimate. This is reflected in the EKF adapting the influence of the measurement on the final composition estimate by altering its corresponding variance. During an experiment, the variance of the curvature methods is augmented by the least squares fit, i.e., the normalized least-squares residual SS_{norm}:

$$SS_{norm} = \frac{SS_{res}}{SS_{tot}} \qquad \text{(Equation 14)}$$

SS_{res} is the residual sum of squares obtained from the least squares and SS_{tot} is the total sum of squares that is proportional to the variance of the data. The further SS_{norm} is away from 0 and the closer it is to 1, the worse the least-square fit, and intermediate estimates $\widehat{P}_{od}[k_h]$, $\widehat{E}_{od}[k_h]$ and $\widehat{P}_{fl}[k_h]$ are trusted less. This is based on the correlation observed between the SS_{norm} and the estimation error. This metric of confidence in the estimates captures both estimation errors through temperature change as well as those arising due to measurement noise, as both affect the quality of the least squares fit.

The remaining quality limitations are not captured by the residual and thus are separately taken into account by increasing the individual uncertainty at the lower and higher temperature range or around the critical temperature. The uncertainties of the five measurements and estimates are reflected in the resulting time-varying measurement uncertainty matrix $R[k]$.

Determining bacteria growth rates

The derivation of the bacteria growth rates is demonstrated here with *P. putida* but can be performed equivalently with *E. coli*. The growth rates were determined by growing the bacteria in monocultures, such that the OD measurements could be directly used to track bacteria abundance, e.g., $od[k] = P[k]$.

Between dilutions, i.e., in the growth period, the solution of the ODE in Equation 1 equals:

$$P(t) = P(t_l)e^{\mu_P(t-t_l)} \qquad \text{(Equation 15)}$$

where t_l corresponded to the point of time right after a dilution period. In the short time frame between dilutions, the growth rate μ_P was assumed to be constant. Instead of fitting μ_P to the exponential in Equation 15, the latter is transformed to obtain a first-order polynomial. Taking the natural logarithm and discretization leads to:

$$\begin{aligned} \ln P[k] &= \mu_P(t[k] - t[k_l]) + \ln P[k_l] \\ &= \mu_P t[k] + \ln P[k_l] - \mu_P t[k_l] \\ &= \mu_P t[k] + c \end{aligned} \qquad \text{(Equation 16)}$$

In the next step a line can be fitted through all data points k in the growth period. To account for the transformation and to obtain the optimal least-squares fit for the exponential function, the data points were weighted with $P[k]$. Finally, the growth rate was acquired by reading out the gradient of the fitted line.

Determining pyoverdine production rates

The Pyoverdine production rate μ_F can be determined by rearranging Equation 13:

$$\begin{pmatrix} \frac{P[k_h]}{\mu_P}\left(e^{\mu_P(t[k_l]-t[k_h])}-1\right) \\ \vdots \\ \frac{P[k_h]}{\mu_P}\left(e^{\mu_P(t[k_h]-t[k_h])}-1\right) \end{pmatrix} \mu_F = \begin{pmatrix} F[k_l]-F[k_h] \\ \vdots \\ F[k_h]-F[k_h] \end{pmatrix} \quad \text{(Equation 17)}$$

where μ_P and $P[k_h]$ were obtained for each growth period as described above, and growth and production rates were assumed to be constant for the short time between dilutions. Applying an unweighted least-squares minimization gives the production rate μ_F that optimally fits the observed data in the growth period.

Time-varying measurement uncertainty matrix

In the measurement update step, five different updates were formulated. This includes the measurements *od* and *fl* as well as the intermediate estimates $\widehat{P}_{od}$, $\widehat{E}_{od}$, and $\widehat{P}_{fl}$. These can be compiled in the measurement vector $\mathbf{y}_{full}[k]$:

$$\mathbf{y}_{full}[k] = \left(od[k] \quad fl[k] \quad \widehat{P}_{od}[k] \quad \widehat{E}_{od}[k] \quad \widehat{P}_{fl}[k] \right)^T \quad \text{(Equation 18)}$$

The measurement uncertainty of $\mathbf{y}_{full}$ was then summarized in the matrix $\mathbf{R}_{full}[k]$ that got utilized in the measurement update step of the EKF:

$$\begin{aligned} \mathbf{R}_{full}[k] = diag(&\sigma_{od}, \\ &f_1(\sigma_{fl}, T[k]), \\ &f_2(\sigma_{P,od}, \mu_P[k], \mu_E[k], SS_{norm,od}[k]), \\ &f_2(\sigma_{E,od}, \mu_P[k], \mu_E[k], SS_{norm,od}[k]), \\ &f_3(\sigma_{P,fl}, T[k], SS_{norm,fl}[k])) \end{aligned} \quad \text{(Equation 19)}$$

Note that during cycles where the intermediate estimates are not available, $\mathbf{y}_{full}$ and $\mathbf{R}_{full}$ are reduced to:

$$\mathbf{y}[k] = \left(od[k] \quad fl[k] \right)^T \quad \mathbf{R}[k] = diag\left(\sigma_{od}, f_1(\sigma_{fl}, T[k])\right) \quad \text{(Equation 20)}$$

QUANTIFICATION AND STATISTICAL ANALYSIS

This study reports our mathematical model for estimating bacterial growth, with the details summarised in the above method details section.

Cell Reports Methods, Volume *5*

Supplemental information

Directing microbial co-culture composition using cybernetic control

Ting An Lee, Jan Morlock, John Allan, and Harrison Steel

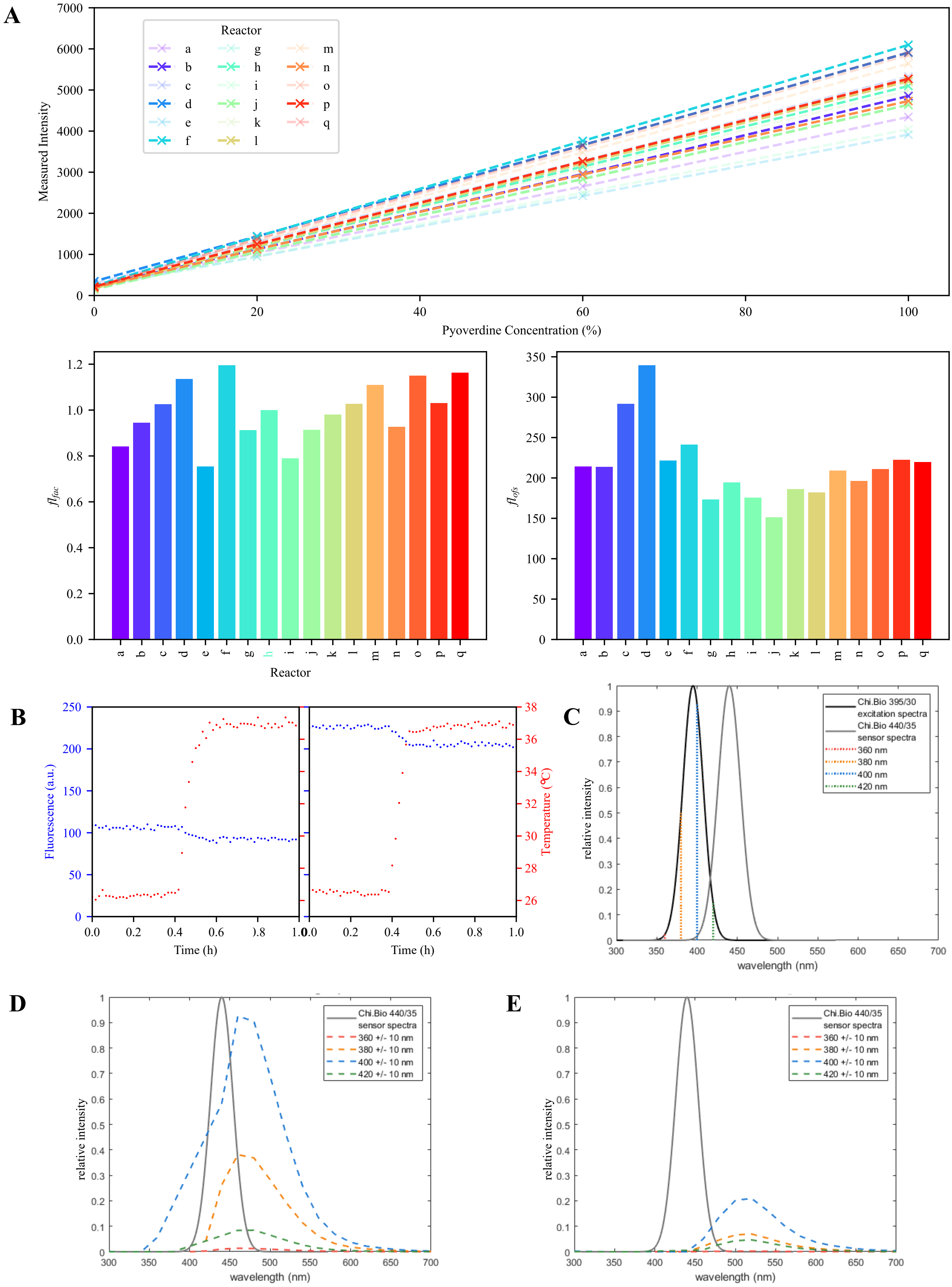
A
Reactor
a
b
c
d
e
f
g
h
i
j
k
l
m
n
o
p
q
Measured Intensity
Pyoverdine Concentration (%)
fl_{fac}
fl_{ofs}
Reactor
B
Fluorescence (a.u.)
Temperature (°C)
Time (h)
Time (h)
C
Chi.Bio 395/30 excitation spectra
Chi.Bio 440/35 sensor spectra
360 nm
380 nm
400 nm
420 nm
relative intensity
wavelength (nm)
D
Chi.Bio 440/35 sensor spectra
360 +/- 10 nm
380 +/- 10 nm
400 +/- 10 nm
420 +/- 10 nm
relative intensity
wavelength (nm)
E
Chi.Bio 440/35 sensor spectra
360 +/- 10 nm
380 +/- 10 nm
400 +/- 10 nm
420 +/- 10 nm
relative intensity
wavelength (nm)

Figure S1: Bioreactor measurements for composition information, related to Figure 2. (A) Calibrating fluorescence readings in different bioreactors. Each reactor was given a unique identifier and then calibrated (top) by measuring fluorescence (a.u.) of a serial dilution of a filter-sterilised *Pseudomonas putida* (*P. putida*) monoculture (removing cells but maintaining fluorescent extracellular pyoverdine). Used excitation/excitation wavelengths of 395/$440\,\mathrm{nm}$ and diluted with M9 media. These measurements were used to calculate a factor fl_{fac} (bottom left) and offset fl_{ofs} (bottom right) for each reactor, where the factor is relative to reactor h. **(B) Effect of temperature on media and pyoverdine.** Reactor runs of just fresh media (M9 + casamino acid (CAA), left) and a filtered *P. putida* monoculture (i.e. M9 + CAA + pyoverdine, right). The negligible ($<$30 a.u.) change in fluorescence across a temperature range larger than conditions used in experiments for both plain media and media with pyoverdine indicates that large changes in fluorescence observed in experiments with cells are due to changes in pyoverdine production. **(C) Fluorescence overlap accounting for hardware - measuring hardware spectra** Fluorescence overlap remains minimal even when taking into account the spectra of the Chi.Bio's 395 nm excitation LED, which has a full width half maximum (FWHM) of 30 nm, and the spectra of the 440 nm sensor, which has a FWHM of 35 nm. (Black curve) relative intensity of different wavelengths of light emitted by the Chi.Bio when the 395/30 nm laser is turned on, and (grey curve) relative intensity of wavelengths measured by the Chi.Bio's 440/35 nm sensor. **(D) Fluorescence overlap accounting for hardware - *P. putida*** Emission spectra of *P. putida* excited with different wavelengths of light (360, 380, 400, 420 nm) measured in a plate reader with a step size and bandwidth of 20 nm, scaled according to the intensity of different excitation wavelengths that result from turning on the 395/30 nm laser in A. The areas under the intersection of the Chi.Bio440/35 sensor spectra (grey curve) with the emission spectra thus represent the fluorescence of *P. putida* measured when turning on the 395/30 nm laser. **(E) Fluorescence overlap accounting for hardware - *Escherichia coli* (*E. coli*)** Same as D, but for *E. coli* (scaled emission spectra when excited at wavelengths of 360, 380, 400, 420 nm).

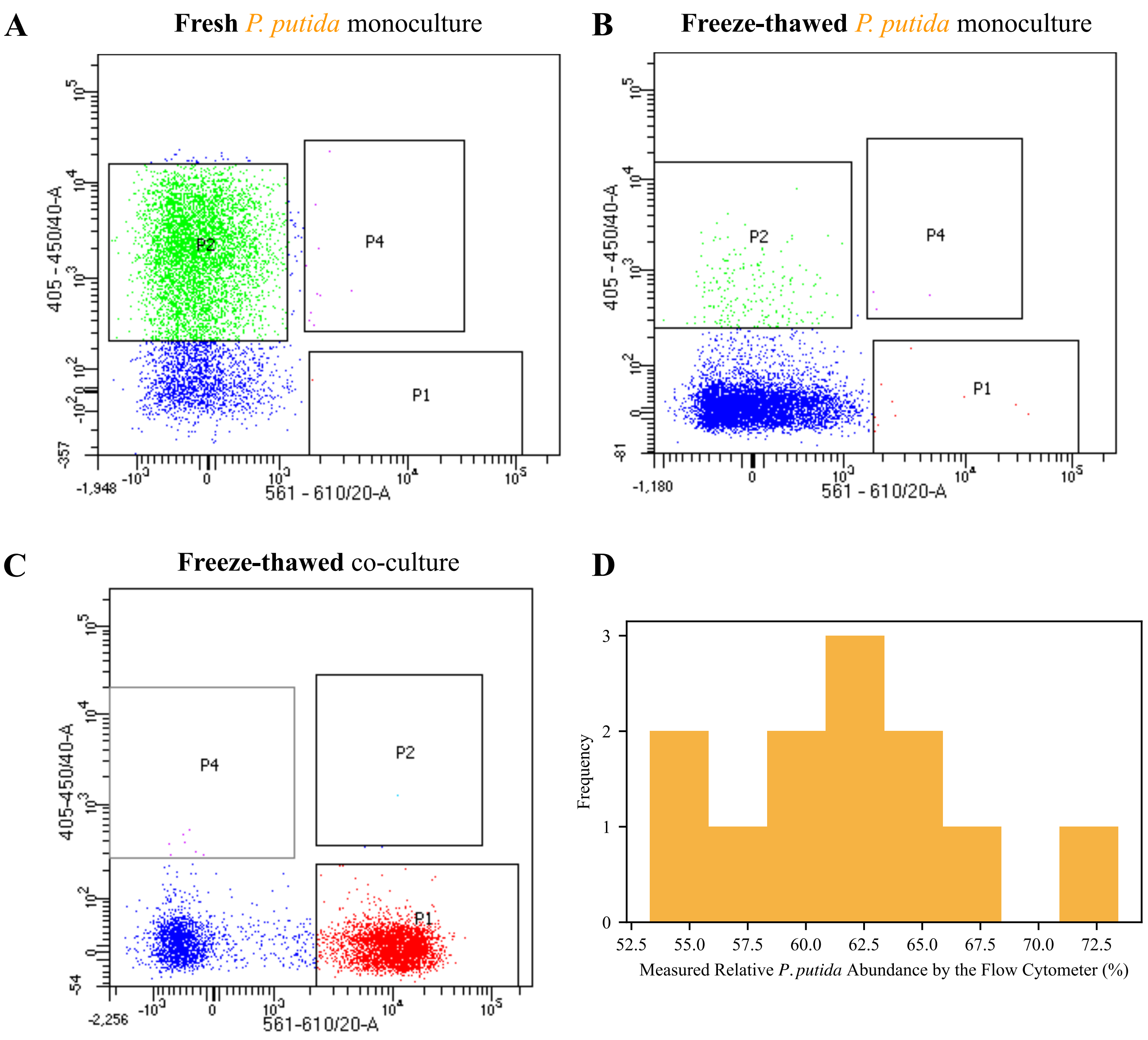

Figure S2: flow cytometry data, related to STAR Methods. **Flow cytometry data.** Freeze-thawing stored samples causes pyoverdine to leak out of cells. Flow cytometry gating of **fresh (A)/freeze-thawed (B) samples** from a *P. putida* monoculture, and a **freeze-thawed sample from a co-culture (C)**. Cells were first gated by forward and side scatter (not shown), and then by fluorescence intensity for pyoverdine (405 - 450/40-A) or red fluorescent protein (RFP) (561 - 610/20-A) wavelengths. Fresh *P. putida* has significant amounts of pyoverdine fluorescence, but almost is lost after freeze-thawing. In the flow cytometer, species are instead distinguished using the RFP's fluorescence, which persists after a freeze-thaw. **(D) Flow cytometer noise.** Histogram of twelve flow cytometer measurements of the same composition. This resulted in an estimated flow cytometry measurement uncertainty with a standard deviation of $5.3\,\%$.

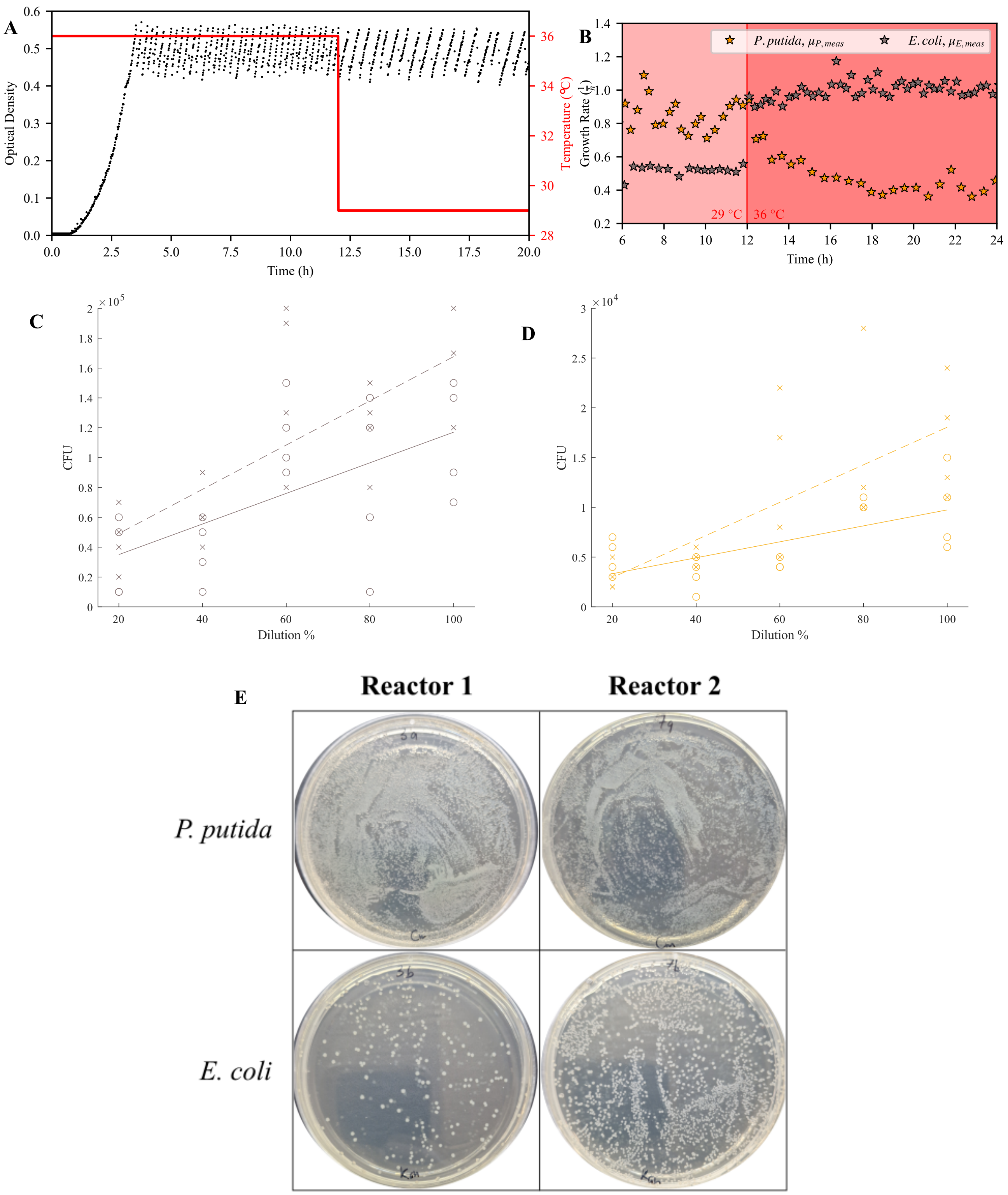
A
Optical Density
Temperature (ºC)
Time (h)
B
Growth Rate (1/h)
P. putida, $\mu_{P,meas}$
E. coli, $\mu_{E,meas}$
29 °C
36 °C
Time (h)
C
×10^5
CFU
Dilution %
D
×10^4
CFU
Dilution %
E
Reactor 1
Reactor 2
P. putida
E. coli

Figure S3: growth dynamics and composition measurements, related to Figure 2. (A) OD dithering around setpoint. The total optical density (OD) (measured at 600_{nm}) of the culture dithers around a setpoint of 0.5, growing from 0 until it reaches 0.545, at which point fresh media is pumped in until the OD is diluted down to 0.455. This creates phases of dilution and growth from which we are able to calculate the growth rate of the mono or co-culture). When the temperature changes, the steepness of the curve during the growth period visibly changes as well. **(B) Change of the growth rates under a temperature change from $29\,^{\circ}\mathrm{C}$ to $36\,^{\circ}\mathrm{C}$.** Growth rates of *P. putida* and *E. coli* calculated from monocultures OD data, where dark red background = $36\,^{\circ}\mathrm{C}$, light red background = $29\,^{\circ}\mathrm{C}$. *P. putida* has a laggy response to temperature change in both directions. **Number of Colony Foming Units (CFUs) from a dilution series of *E. coli* (C) and *P. putida* (D) from bioreactor monoculture**. Diluted with $20\,\%$ intervals using PBS. Technical replicates at each dilution and biological replicates in different reactors vary, possibly due to noise in OD measurement. Flow cytometry, which measures ratios, is unaffected by this type of noise. **(E) Composition of biofilms.** Biofilms from a *P. putida* KT2440 (Cm resistance) and *E. coli* (Km resistance) co-cultures mixed at a 1:1 ratio and maintained at the 33.2C critical temperature. The biofilm in the reactors after overnight growth were scraped, resuspended in 100 ul PBS, and then spread onto plates with chloramphenicol or kanamycin. In both reactors, the chloramphenicol plates have semi-confluent growth, while the kanamycin plates have fewer colonies, indicating that the biofilm contains both species but consists primarily of *P. putida* colony forming units. Quantitative comparison *between* reactors is avoided, as the biofilm thickness and amount scraped was similar but not standardised. Controls of *P. putida* on kanamycin plates or *E. coli* on chloramphenicol plates have no colonies (not shown). Co-cultures with *P. putida* -lapA grown at the same time did not have visible biofilm, in line with monoculture behaviour.

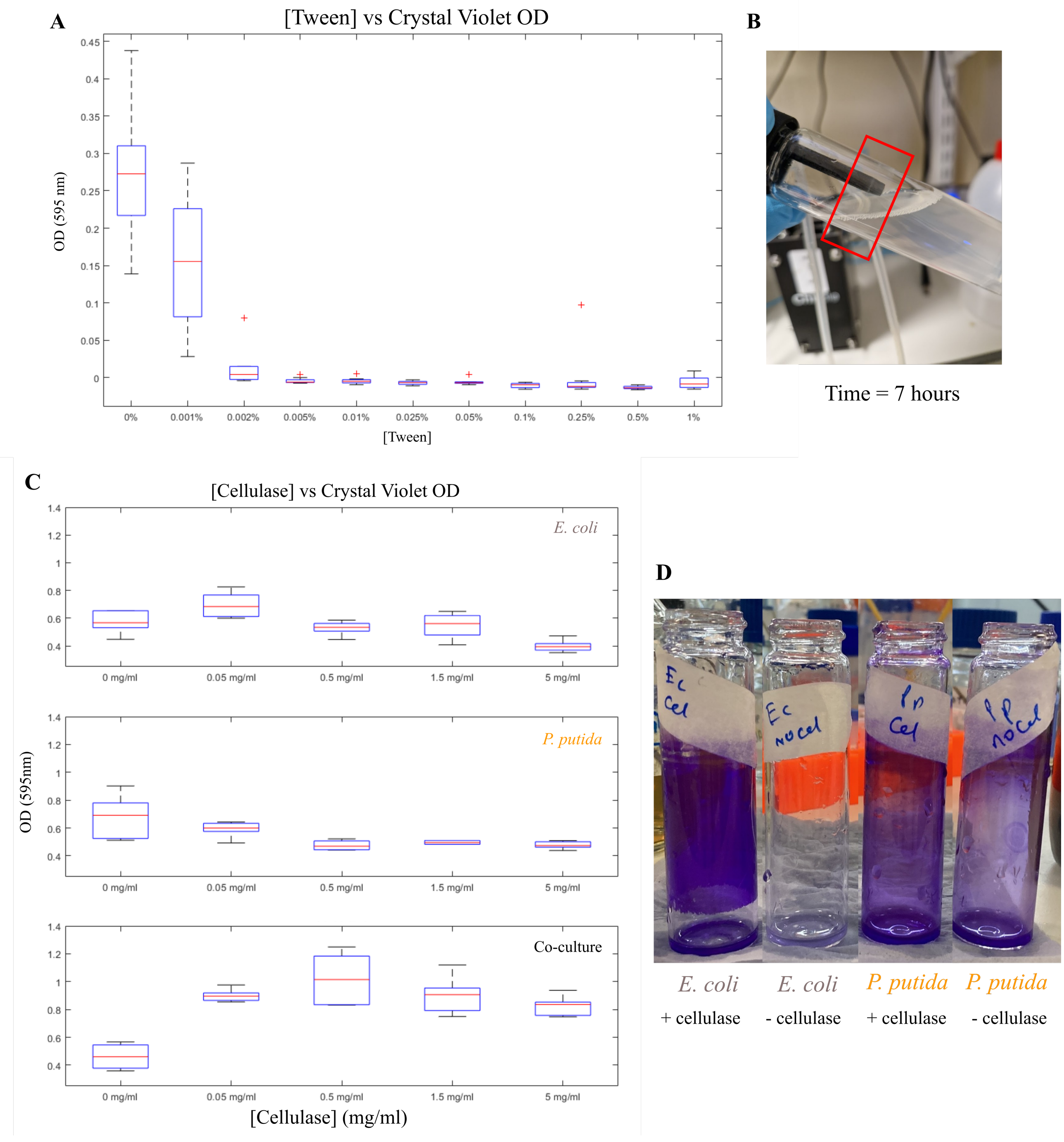
A
[Tween] vs Crystal Violet OD
OD (595 nm)
0.45
0.4
0.35
0.3
0.25
0.2
0.15
0.1
0.05
0
0%
0.001%
0.002%
0.005%
0.01%
0.025%
0.05%
0.1%
0.25%
0.5%
1%
[Tween]
B
Time = 7 hours
C
[Cellulase] vs Crystal Violet OD
E. coli
P. putida
Co-culture
OD (595nm)
1.4
1.2
1
0.8
0.6
0.4
0 mg/ml
0.05 mg/ml
0.5 mg/ml
1.5 mg/ml
5 mg/ml
[Cellulase] (mg/ml)
D
E. coli
+ cellulase
E. coli
- cellulase
P. putida
+ cellulase
P. putida
- cellulase

Figure S4: anti-biofilm measures, related to Figure 3 Polysorbate (Tween) 20 to prevent biofilms in 96 well plates. Range of concentrations of Tween 20 vs OD_{595} of ethanol-solubilised crystal violet used to stain the wells of 96 well plate growing *P. putida*. A higher OD = more biofilm. Compared to the no-Tween control, concentrations $>0.001\,\%$ have significantly less biofilm formation in plates. **(B) Polysorbate (Tween) 20 to prevent biofilms in bioreactor.** Monoculture of *P. putida* in a reactor vial after 7 hours of growth with $0.01\,\%$ Tween 20. A thin biofilm has still formed after 7 hours at the air-water interface, as highlighted in the red box. **(C) Cellulase to prevent biofilms in 96 well plates.** Range of concentrations of cellulase vs OD_{595} of ethanol-solubilised crystal violet used to stain the wells of 96 well plates growing *E. coli* (top), *P. putida* (middle), or both (bottom). A higher OD = more biofilm. Concentrations above $0.5\,\mathrm{mg\,mL^{-1}}$ had less *P. putida* biofilm in plates, while cellulase did not have a clear effect on *E. coli*. Co-cultures had more biofilm when cellulase was added, but not in a concentration-dependent way. **(D) Cellulase to prevent biofilms in bioreactor.** Monocultures grown in a reactor vial overnight with/without $0.5\,\mathrm{mg\,mL^{-1}}$ cellulase, where depth of purple indicates amount of biofilm. Cellulase caused *E. coli* to form an extremely thick biofilm, while it appeared to slightly inhibit *P. putida* biofilm formation.

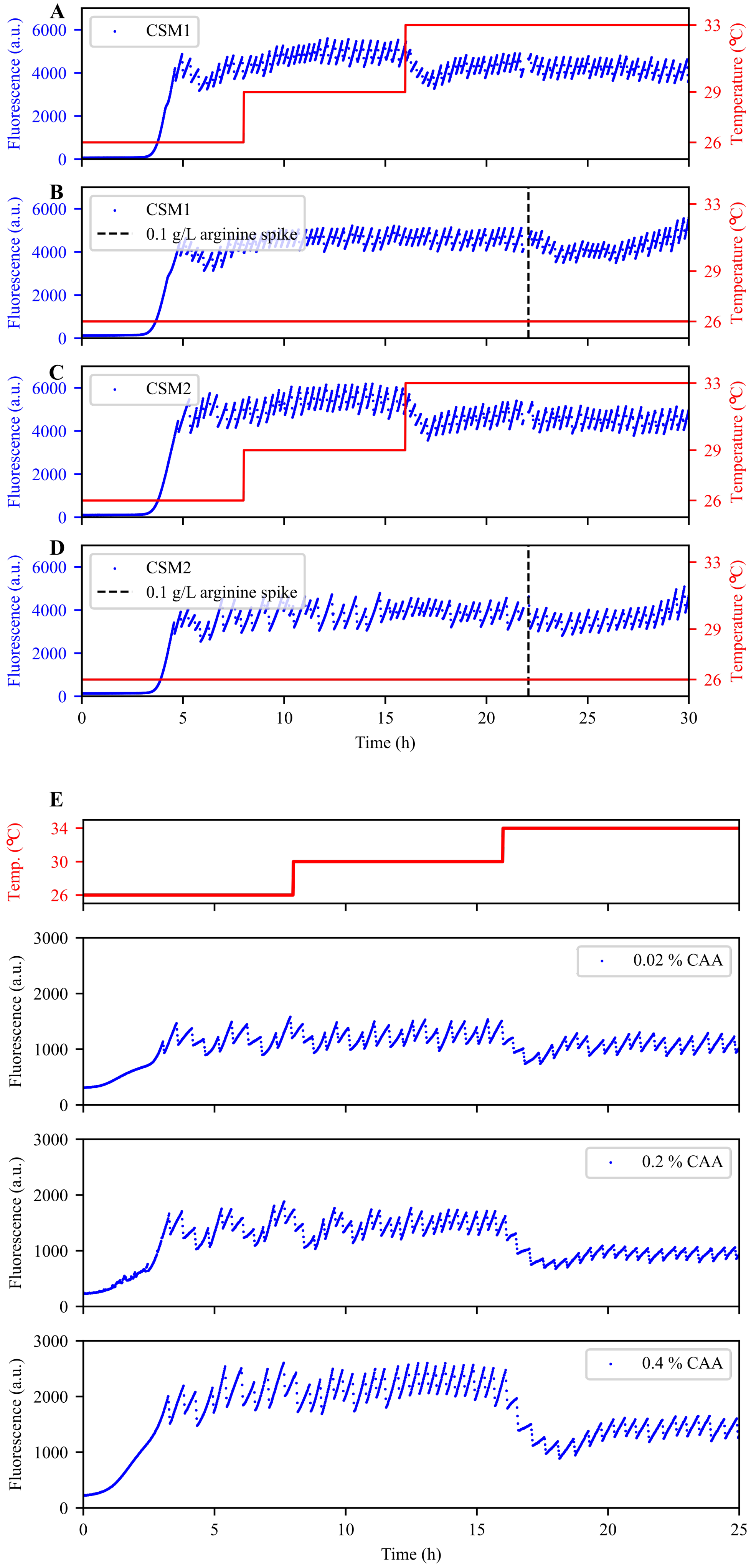
A
CSM1
B
CSM1
0.1 g/L arginine spike
C
CSM2
D
CSM2
0.1 g/L arginine spike
Fluorescence (a.u.)
Temperature (℃)
0
2000
4000
6000
26
29
33
0
5
10
15
20
25
30
Time (h)
E
Temp. (℃)
26
30
34
0.02 % CAA
0.2 % CAA
0.4 % CAA
0
1000
2000
3000
Time (h)

Figure S5: effect of casamino acids (CAA) and complete synthetic amino acid mixtures (CSM) on oscillations, related to Figure 3. (A - D) Effect of different CSMs and arginine on oscillating fluorescence *P. putida* monocultures grown with double dropout Complete Synthetic Mixtures (- arginine and -tryptophan, both thought to influence pyoverdine production, from Formedium (see Key Resources Table). CSM1 (A, B) is supplemented with $100\,\mathrm{mg\,L^{-1}}$ serine, while CSM2 (C, D) is supplemented with $100\,\mathrm{mg\,L^{-1}}$ serine and glutamine. Runs B and D were spiked with $100\,\mathrm{mg\,L^{-1}}$ arginine at around time = 22 hours, which caused production to briefly drop before rising. **Effect of temperature on oscillatory behaviour of pyoverdine production.** Fluorescence is affected by the dithering behaviour of the culture (spiky increases/drops as the culture grows/is diluted)- this is not seen in the Fig. 3D/E, which were grown without dithering (i.e. very small dilutions whenever OD exceeded the setpoint, leading to an almost constant OD). Aside from the spikes, oscillations are also visible at $26\,^{\circ}\mathrm{C}$. The amplitude of the oscillations appears to be related to temperature, decreasing at warmer temperatures.

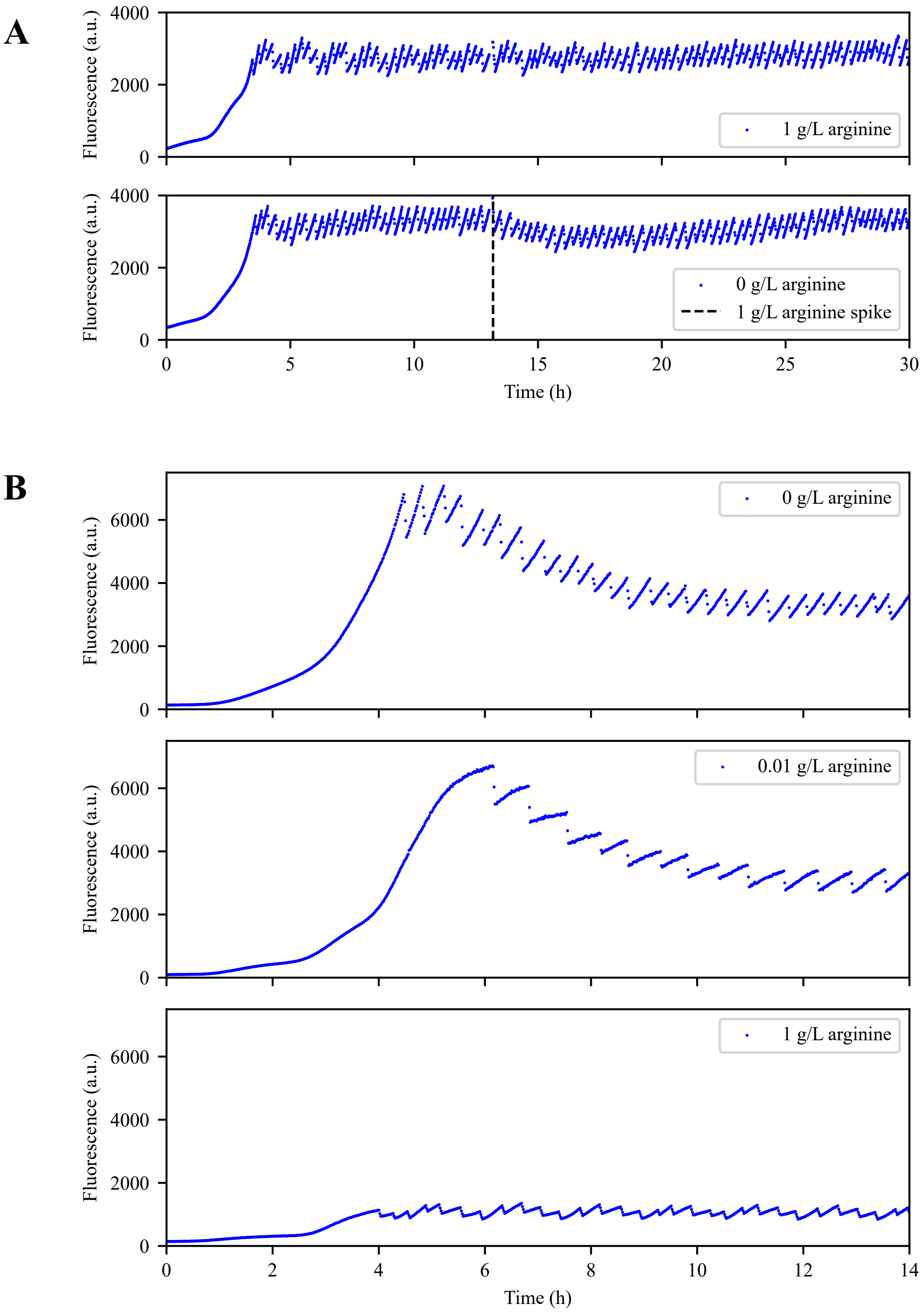
A
B
Fluorescence (a.u.)
Time (h)
1 g/L arginine
0 g/L arginine
1 g/L arginine spike
0.01 g/L arginine
0
2000
4000
6000
5
10
15
20
25
30
2
4
6
8
12
14

Figure S6: effect of arginine on pyoverdine production, related to Figure 3. (A) Effect of arginine with complex amino acid mixtures on oscillatory behaviour of pyoverdine production. *P. putida* monocultures grown with $0.2\,\%$ CAA and $1\,\mathrm{g\,L^{-1}}$ arginine (top) still has dampened oscillatory behaviour at $29\,°\mathrm{C}$. When grown with $0.2\,\%$ CAA but no arginine, and then spiked with $1\,\mathrm{g\,L^{-1}}$ arginine at around time = 13 hours (bottom), pyoverdine production drops briefly before rising slowly. **(B) Effect of arginine with simple amino acid mixtures on oscillatory behaviour of pyoverdine production.** *P. putida* monocultures grown with $3\,\mathrm{g\,L^{-1}}$ serine and $0.5\,\mathrm{g\,L^{-1}}$ glutamine, using values suggested from Maser et al.[1] along with a range of arginine concentrations. Arginine has a clear effect, with lower concentrations appearing to dampen oscillations but cause a peak in pyoverdine before settling. Excess arginine creates oscillations that did not dampen after 15 hours.

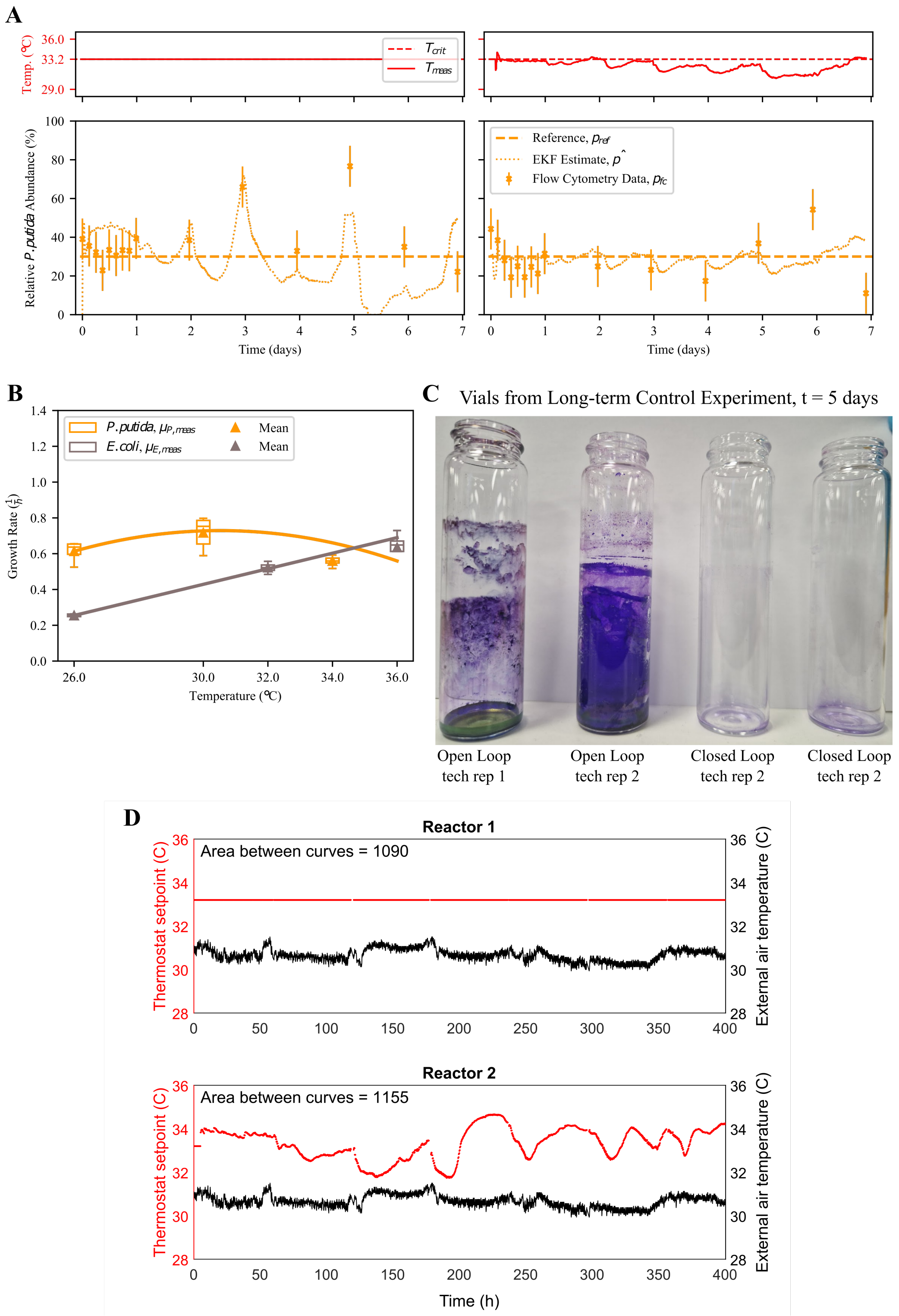
A
Temp. (℃)
36.0
33.2
29.0
T_{crit}
T_{meas}
Relative P. putida Abundance (%)
Reference, p_{ref}
EKF Estimate, $\hat{p}$
Flow Cytometry Data, p_{fc}
Time (days)
B
Growth Rate ($\frac{1}{h}$)
P.putida, $\mu_{P,meas}$
E.coli, $\mu_{E,meas}$
Mean
Mean
Temperature (℃)
C
Vials from Long-term Control Experiment, t = 5 days
Open Loop tech rep 1
Open Loop tech rep 2
Closed Loop tech rep 2
Closed Loop tech rep 2
D
Reactor 1
Area between curves = 1090
Reactor 2
Area between curves = 1155
Thermostat setpoint (C)
External air temperature (C)
Time (h)

Figure S7: long term co-culture control, related to Figure 7. (A) Additional replicates of long-term control. Similar trends are observed to replicates from Fig. 6, where open-loop control maintains the composition around the setpoint for a day, but then experiences large fluctuations over the rest of the week. The closed-loop control is still around the setpoint by the end of the experiment, though the *P. putida* abundance rose to around $50\,\%$ on the second last day. **(A) Effect of CAA concentration on growth rate.** Growth rate of monocultures cultured at a range of CAA concentrations. Because *E. coli* is much more affected by decreasing [CAA] than *P. putida*, the critical temperature is increased and there is a smaller temperature window to select for *E. coli*. The smaller difference in relative growth rate means that the system would respond slower to control inputs. **(C) Vials from the long-term co-culture control experiments, t=5 days.** Vials are stained with crystal violet, depth of purple represents amount of biofilm. Open-loop technical replicates had no controller, but the media temperature was set to the critical temperature. Closed-loop technical replicates uses the controller. Closed-loop vials have significantly less biofilm, presumably because the *P. putida* population was maintained at around $30\,\%$. **(D) Energy expenditure needed for control.** Temperature of the thermostat (i.e. temperature that the reactor maintained) in red for a reactor kept at a constant temperature (top) or a fluctuating temperature (bottom) for control to maintain a static composition from Fig. 7. The ambient temperature of the air outside the reactor is in black. The power used by the reactor to heat the culture is linearly proportional to the temperature difference, and hence total energy expended is (approximately) proportional to the area between the thermostat and external air temperature curves. In the reactor with feedback control, periods of heating above the setpoint would draw additional power, but periods of cooling below the setpoint draw none. The relative area between the curves is 1090 for a static setpoint and 1155 for feedback control, representing a 6% increase in energy expenditure to achieve compositional control.

References

1. Maser, A., Peebo, K., Vilu, R., and Nahku, R. (2020). Amino acids are key substrates to *Escherichia coli* BW25113 for achieving high specific growth rate. Research in Microbiology *171*, 185–193. URL: `https://www.sciencedirect.com/science/article/pii/S0923250820300176`. doi: 10.1016/j.resmic.2020.02.001.
